# Safety evaluation of genipin in Beagle dogs: acute and 28-day repeated oral toxicity studies

**DOI:** 10.3389/fphar.2026.1924890

**Published:** 2026-10-30

**Authors:** Qianchao Meng, Wei Sun, Lijing Kang, Mingshu Huang, Taofen Wu, Yingmin Gu, Jianqiang Zhang, Zhili Cao, Fei Yang, Guangtao Yao

**Affiliations:** Center for Drug Safety Evaluation and Research, Shanghai University of Traditional Chinese Medicine, Shanghai, China

**Keywords:** acute toxicity, genipin, safety evaluation, subacute toxicity, target organ toxicity

## Abstract

**Introduction:**

Genipin, a typical iridoid constituent derived from the fruits of *Gardenia jasminoides* Ellis, is an essential *in vivo* metabolite of geniposide. It exhibits diverse potent biofunctions, including hepatoprotection, anti-inflammation, neuroprotection, antioxidation, and glycolipid metabolism modulation. Nevertheless, the systemic toxicological profile of genipin remains insufficiently characterized, with particularly limited safety evidence from non-rodent models. This study aimed to evaluate the acute and subacute oral toxicity of genipin in Beagle dogs as a preclinical safety assessment.

**Methods:**

Acute toxicity was assessed using single oral doses of genipin at 29, 41, 59, 84, 120, and 140 mg/kg. For subacute toxicity, repeated-dose groups were administered 3, 9, and 27 mg/kg/day for 28 days. Clinical signs, body weight, food intake, hematology, clinical chemistry, urinalysis, electrocardiography, fecal occult blood, and histopathology were evaluated.

**Results:**

The results showed that the oral approximate lethal dose of genipin was 120.0–140.0 mg/kg, with doses ≥41 mg/kg inducing thymic, hepatorenal, and gallbladder toxicity. Repeated 27 mg/kg/day genipin caused gastrointestinal irritation, anemia, prolonged coagulation parameters, and toxicity to the hepatobiliary system, thymus, bone marrow, and gonads; 3 and 9 mg/kg/day genipin induced mild male gonad toxicity. Bone marrow toxicity and gastric mucosal irritation were fully reversible after recovery, while hepatic, thymic, and gonadal toxicity showed partial recovery.

**Conclusion:**

This study clarified the dose-toxicity relationship, major target organs, and toxic reversibility of genipin following oral exposure in non-rodent species. These findings provide robust preclinical toxicological evidence to support rational dosage optimization, risk assessment, and the safe use of genipin.

## 1 Introduction

Genipin is a characteristic iridoid constituent isolated from the dried ripe fruits of *Gardenia jasminoides* Ellis (Rubiaceae) (Sobolewska et al., 2025). Genipin possesses prominent hepatoprotective (Seo et al., 2017; Wang et al., 2019), anti-inflammatory (Fan et al., 2020), neuroprotective (Isabel et al., 2022), antioxidant (Li et al., 2026; Deng et al., 2025), and anticancer (Wu J. et al., 2023) effects. Additionally, genipin has been demonstrated to have anti-infarction (Franke et al., 2021) and hypoglycemic effects (Wu Y. et al., 2023), further enriching the pharmacological significance of *Gardenia jasminoides* in ethnomedicine.

Genipin also possesses unique inherent biological and physicochemical properties. As a multifunctional natural compound, the applications of genipin have expanded across food science (Liu et al., 2022; Xu et al., 2024), biomedicine (Bryś et al., 2022), and material engineering (García-García et al., 2025; Tolabi et al., 2025) due to its excellent biocompatibility, superior biodegradability, and stable crosslinking. In food science, its crosslinked films and hydrogels enhance fruit preservation and active-packaging functionality. In biomedicine, genipin-crosslinked hydrogels are used in drug delivery systems, including natural, chemical, and protein-based delivery. The genipin-crosslinked hydrogel network facilitates long-term *in vivo* drug delivery. It serves as a component of controlled-release systems, enabling site-specific, time-controlled drug release to improve efficacy and reduce side effects. In tissue engineering, genipin, as a crosslinker, enhances the structural stability and functionality of biomaterials. Previous studies have successfully combined genipin with other biomaterials to promote bone regeneration (Cheon et al., 2021). Genipin-crosslinked hydrogels are also utilized in wound dressings and wound closure (Andrade et al., 2022; Hu et al., 2023). Genipin also serves as a precursor of natural dietary blue pigments, widely applied in beverages, confections, and processed foods. It enables the multi-field transformation of *Gardenia jasminoides* from a single medicinal application to diversified utilizations in medicine, the food industry, and biomaterials. Accordingly, genipin provides critical material support for modern innovation and high-value utilization of traditional ethnomedicinal herbs, representing a typical paradigm for translational research on ethnopharmacological resources. Given genipin’s prominent pharmacological activities and ethnomedicinal value, a comprehensive elucidation of its safety profile after oral administration is highly significant.

With the transition of genipin from traditional herbal use to large-scale industrial applications, human exposure frequency and dosage have significantly increased, raising urgent concerns about its toxicity. Animal studies have demonstrated that genipin induces dose-dependent hepatotoxicity (Luo et al., 2021), which is associated with the inhibition of UDP-glucuronosyltransferase and cytochrome P450 enzyme activities (Wang et al., 2023), indicating definite toxicological risks upon systemic exposure. However, current toxicological data are predominantly derived from murine experiments, *in vitro* cell assays, and biomaterial implantation models, while systematic oral toxicity data from non-rodent species remain extremely limited (Tian et al., 2022). As a well-recognized classic non-rodent model for regulatory toxicology, laboratory Beagles (*Canis lupus familiaris*, Beagle dogs) share high anatomical, physiological, and metabolic similarities with humans, enabling the acquisition of unique data concerning target organ toxicity, dose tolerance, toxic reversibility, and translational safety that rodent studies cannot fully reflect. Therefore, investigating the acute and 28-day subacute oral toxicity of genipin in Beagle dogs is essential to establish a comprehensive and systematic preclinical safety evaluation system for genipin. From an ethnopharmacological perspective, the present findings provide critical scientific evidence for the safety assessment of *Gardenia jasminoides*-derived bioactive components and traditional preparations containing genipin or its precursors. This study addressed whether oral genipin could induce toxicity in non-rodent species, including its primary target organs and the reversibility of toxic manifestations.

In this study, we used acute and 28-day repeated oral toxicity tests to evaluate genipin toxicity in Beagle dogs. The results clarified the dose-toxicity relationship, major target organs, and toxic reversibility of genipin following oral exposure in non-rodent species. These findings provide robust preclinical toxicological evidence to support rational dosage optimization, risk assessment, and the safe utilization of genipin.

## 2 Materials and methods

This study was conducted in accordance with the Good Laboratory Practice (GLP) of the Organization for Economic Cooperation and Development (OECD) and the Technical Guidelines for Pharmaceutical Research published by the National Medical Products Administration (NMPA) of China.

### 2.1 Materials

Genipin (Lot No. zs2010042602) was provided by the Teaching and Experiment Center of Shanghai University of Traditional Chinese Medicine (SHUTCM), China. The purity of genipin was greater than 95%. Enteric-coated gelatin hollow capsules (Lot No. 0733409), used as a control, were provided by Anhui Huangshan Capsule Co., Ltd., China. Enteric-coated gelatin genipin capsules were prepared by filling genipin powder into enteric-coated gelatin hollow capsules. All clinical biochemical reagents were purchased from SHINO-TEST CORPORATION (Tokyo, Japan) unless otherwise stated. All hematological reagents were purchased from Shanghai Bailai Biological Products Co., Ltd. (Shanghai, China) unless otherwise stated. All coagulation reagents were purchased from Siemens Healthcare Diagnostics Products GmbH (Marburg, Germany) unless otherwise stated.

### 2.2 Animals

Conventional laboratory Beagles (*C. lupus familiaris*, Beagle dogs), age 7∼9 months, weight 6.9∼9.1 kg, were purchased from Beijing Marshall Biotechnology Co., Ltd. (Beijing, China) and housed individually in stainless steel cages under controlled environmental conditions (temperature: 16 °C–26 °C, relative humidity: 40%–70%, 12 h light/dark cycle, ventilation 8-10 times/hour). Beagle dogs were fed a dog diet once daily and had free access to tap water. Animal treatment protocols were reviewed and approved by the Experimental Animal Ethics Committee of SHUTCM.

### 2.3 Acute oral toxicity study in Beagle dogs

The study followed the Guideline for Acute Toxicity Tests of Traditional Chinese Medicines and Natural Products published by the NMPA.

Beagle dogs were fasted overnight, and genipin was administered via single oral gavage at doses of 29, 41, 59, 84, 120, and 140 mg/kg, with one dog assigned to each dose group. The no-observed-adverse-effect level (NOAEL), minimum lethal dose (MLD), maximum non-lethal dose (MNLD), and approximate lethal dose were determined. Empty enteric-coated gelatin hollow capsules were administered as the control. All dogs were monitored daily for 15 days after administration.

During the experimental period, clinical signs and food intake were recorded daily. Body weights were measured before administration and at 1, 4, 7, and 15 days after administration. Body temperature, fecal occult blood, and hematology were measured simultaneously, except the 4th day after administration. Lead II electrocardiograms (ECGs) were obtained before administration and at 2 h, 4 h, 7, and 15 days after administration. Clinical biochemistry was measured before administration and at 1, 7, 10, and 15 days after administration. At the end of the observation period, Beagle dogs were anesthetized by intravenous injection of 3% sodium pentobarbital solution (30 mg/kg body weight). Adequate anesthesia was confirmed by the absence of toe-pinch, corneal, and palpebral reflexes. Bilateral femoral arteries were exposed and transected to exsanguinate until blood flow ceased. The animals were continuously monitored for at least 5 min to confirm irreversible loss of vital signs, including spontaneous respiration, heartbeat, fixed dilated pupils, and pale mucous membranes. Necropsy was performed immediately after confirmation of death.

### 2.4 28-Day subacute oral toxicity study in Beagle dogs

The 28-day subacute oral toxicity study was conducted in accordance with the technical guidelines for pharmaceutical research published by the NMPA, which are equivalent to the OECD guideline 407: repeated dose 28-day oral toxicity.

Beagle dogs were randomly divided into four groups: a control group and three groups receiving 3, 9, and 27 mg/kg genipin by body weight, with six dogs in each group, consisting of three males and three females. Dogs in the control group were administered empty enteric-coated gelatin hollow capsules. Other dogs were administered enteric-coated gelatin genipin capsules via oral gavage once daily, 7 days per week for 4 weeks. Thereafter, dosing was discontinued, and one dog from each group was randomly selected for observation during a 4-week recovery period. During both the administration and recovery phases, clinical signs were observed twice daily. Food intake was recorded once daily, and body weight was recorded once weekly. Hematology, clinical chemistry, coagulation, fecal occult blood, body temperature, ECGs, and ophthalmic examinations-were conducted before administration, during the 4-week administration period, and at the 2nd and 4th weeks of the recovery phase. At the end of the administration or recovery period, urine was collected for urinalysis. Then, the dogs were intravenously anesthetized with 3% sodium pentobarbital (30 mg/kg), with adequate anesthesia confirmed. Bilateral femoral arteries were exposed and transected for complete exsanguination. The animals were monitored for at least 5 min to confirm irreversible cessation of vital signs. Subsequently, major organs and tissues were necropsied and histopathologically examined.

### 2.5 Hematology and clinical chemistry

Whole blood was collected in EDTA-K_2_ tubes for hematology, including red blood cell count (RBC), hemoglobin (HGB), white blood cell count (WBC), hematocrit (HCT), mean corpuscular volume (MCV), mean corpuscular hemoglobin (MCH), mean corpuscular hemoglobin concentration (MCHC), platelet count (PLT), neutrophil count (NEUT), lymphocyte count (LYMPH), monocyte count (MONO), eosinophil count (EOS), and basophil count (BASO). Hematology was performed using the BAYER ADVIA 120 five-part differential hematology analyzer. (Siemens Healthcare Diagnostics Ltd., Ireland). For coagulation analysis, whole blood was collected in sodium citrate tubes. Coagulation parameters, including prothrombin time (PT), activated partial thromboplastin time (APTT), thrombin time (TT), and fibrinogen (FIB), were detected using the Sysmex CA-530 Automatic Coagulation Analyzer (Sysmex Corporation, Kobe, Japan). Serum separated from coagulated whole blood was analyzed using the Hitachi 7080 Automatic Biochemistry Analyzer (Hitachi Ltd., Japan). The clinical chemistry includes aspartate aminotransferase (AST), alanine aminotransferase (ALT), alkaline phosphatase (ALP), blood urea nitrogen (BUN), creatinine (CREA), glucose (GLU), total bilirubin (TB), creatine kinase (CK), γ-glutamyl transferase (GGT), lactate dehydrogenase (LDH), calcium (Ca^2+^), sodium (Na^+^), potassium (K^+^), and chloride (Cl^−^) ions. Additionally, total protein (TP), albumin (ALB), total cholesterol (CHOL), triglycerides (TG), low-density lipoprotein (LDL), and high-density lipoprotein (HDL).

### 2.6 ECG measurements

Lead II electrocardiograms (ECGs) were recorded using the SP2006 electrocardiogram analysis system (Shanghai Manpu Biotechnology Co., Ltd., China). Procedures were performed in a nonsedated, and standing position. Electrodes were placed on the skin surface using animal ECG clips, and ECG paste was applied to maintain electrical contact. Standard ECG variables, including heart rate, P wave amplitude (Pa), corrected P wave amplitude (P’a), ST segment, T wave, corrected T wave (T′), QRS complex, PR interval, and QT interval, were evaluated (Kurogochi et al., 2024).

### 2.7 Urinalysis

Urine was detected using the Roche UriSys two urine analyzer (Roche Diagnostics, Mannheim, Germany). Parameters included urine appearance, specific gravity (SG), pH value, protein (PRO), glucose (GLU), ketone bodies (KET), leukocytes (LEU), erythrocytes (ERY), urobilinogen (UBG), bilirubin (BIL), and nitrite (NIT). Urine sediment examination was performed to confirm the abnormal results further.

### 2.8 Histopathology

Beagle dogs were subjected to gross necropsy. The brain, pituitary gland, thyroid gland, parathyroid gland, salivary glands, thymus, aorta, heart, trachea, lungs, esophagus, stomach, liver, gallbladder, spleen, pancreas, small intestine, large intestine, lymph nodes, adrenal glands, kidneys, ureters, urinary bladder, eyes, optic nerves, mammary glands, sternum, spinal cord, sciatic nerve, testes, epididymides, prostate, uterus, and ovaries were collected from animals in each group. The eyes, testes, epididymides, and Harderian glands were fixed in Davidson’s solution. All remaining tissues and organs were fixed in 10% neutral-buffered formalin. After 24∼48 h, tissues underwent gradient ethanol dehydration, xylene clearing, and paraffin embedding. Paraffin-embedded blocks were sectioned at 4 μm using a rotary microtome (RM2235, Leica, Germany). The sections were stained with hematoxylin-eosin (HE) and examined under a bright-field microscope (BX51, Olympus, Japan). Histopathological evaluation of all tissue sections was performed by a board-certified senior pathologist (−: no lesion (score = 0); ±: minimal lesion (score = 1); +: mild lesion (score = 2); ++: moderate lesion (score = 3); +++: severe lesion (score = 4)). (Sall et al., 2025). For the repeated-dose toxicity study, the following organs were weighed: heart, liver, spleen, lungs, kidneys, brain, pituitary gland, thymus, adrenal glands, testes, epididymides, uterus, and ovaries. The organ coefficients were calculated.

### 2.9 Statistical analysis

The data were analyzed using IBM SPSS Statistics (Version 27, IBM Corp., NY, USA). All values are presented as mean ± standard deviation. All variables were tested for normality and homogeneity of variance prior to analysis. Data with homogeneous variance were analyzed using an unpaired t-test for two-group comparisons or one-way ANOVA followed by Tukey’s multiple comparison test for multiple-group comparisons. Nonparametric tests were used for non-normally distributed data: the Mann-Whitney U test for two independent groups and the Kruskal–Wallis test followed by Dunn’s multiple comparisons test for multiple groups. The Radit method was used to analyze food intake. p < 0.05 was considered statistically significant.

## 3 Results

### 3.1 Acute oral toxicity study in Beagle dogs

Beagle dogs received genipin by oral administration at different doses to observe acute toxic reactions and mortality (Figure 1A).

**FIGURE 1:**
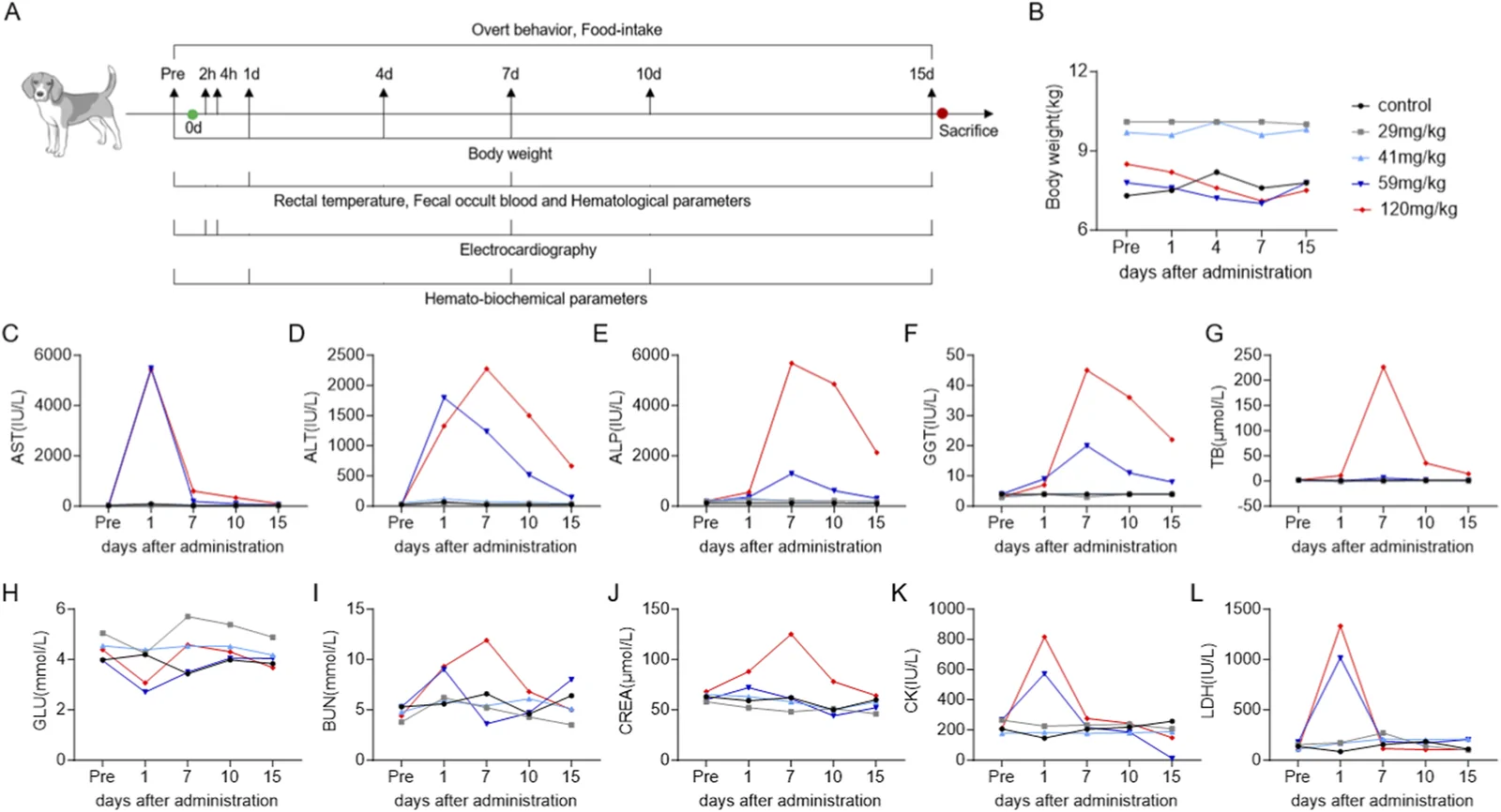
Acute toxicity test results of oral genipin in Beagle dogs. **(A)** Schematic diagram of acute toxicity study of genipin in Beagle dogs. **(B)** Body weight kinetics in genipin-treated Beagle dogs. n = 1. **(C–L)** Time-dependent changes in clinical chemistry, including AST **(C)**, ALT **(D)**, ALP **(E)**, GGT **(F)**, TB **(G)**, GLU **(H)**, BUN **(I)**, CREA **(J)**, CK **(K)**, and LDH **(L)**, after genipin administration. n = 1.

Since dogs in the 84 and 140 mg/kg groups vomited all genipin-loaded capsules following administration, data from these two groups were excluded.

#### 3.1.1 Clinical signs and food intake

Various doses of genipin, except 29 mg/kg, induced varying severities of emesis in Beagle dogs, accompanied by dark green feces and decreased food intake, with a clear dose-dependent relationship (Tables 1, 2).

**TABLE 1:** Clinical signs and Gross necropsy findings in Beagle dogs: Acute oral toxicity of genipin.

Animal no.	Sex	Dose (mg/kg)	Clinical signs	Gross necropsy findings
1	♀	Control	No abnormalities noted	No abnormalities noted
7	♂	29	No abnormalities noted	Smaller thymus size; intestinal mucosal hyperemia
2	♂	41	Emesis of dark green mucous fluid; reduced food intake; dark green feces	Smaller thymus size; intestinal mucosal hyperemia
3	♀	59	Emesis of dark green mucous fluid at 1 and 2 days after administration; reduced food intake, with slight alleviation by day 9; dark green feces	Smaller thymus size; intestinal mucosal hyperemia
5	♀	120	Severe emesis at 2.5 h after administration; dark green mucous fluid; nearly anorexic; lethargy; mental status gradual improvement by day 7 after administration	Smaller thymus size; blue discoloration of bilateral kidneys; distended gallbladder with bile accumulation; focal green discoloration of liver surface; hyperemia of duodenal mucosa with focal ulceration

**TABLE 2:** Food intake during Acute oral toxicity of genipin in Beagle dogs.

Animal no.	Sex	Dose (mg/kg)	Pre	1d	2d	3d	4d	5d	6d	7d	8d	9d	10d	11d	12d	13d	14d
1	♀	Control	-	+	-	-	+	++	-	-	-	-	-	-	-	-	-
7	♂	29	-	+	+	-	-	-	-	+	-	-	-	-	-	-	-
2	♂	41	-	++	++	+	++	++	++	++	+	+	-	+	+	+	+
3	♀	59	-	+++	++	+++	+++	++	++	++	++	+	+	+	+	+	+
5	♀	120	-	+++	+++	+++	+++	+++	+++	++	++	++	++	++	++	++	++

“−”: No residue; “+”: Slight residue; “++”: Moderate residue; “+++”: Complete residue.

#### 3.1.2 Body weight and temperature

Dosing genipin at 59 and 120 mg/kg resulted in decreased body weight in Beagle dogs; the 59 mg/kg group showed complete recovery on day 15, while the 120 mg/kg group did not return to baseline body weight. No obvious decrease was observed in other groups (Figure 1B). Body temperature was normal in all dose groups (data not shown).

#### 3.1.3 Hematology and clinical chemistry

Clinical chemistry was evaluated in Beagle dogs at pre-administration and on days 1, 7, 10, and 15 post-dosing. The results showed that AST and ALT increased in the 59 and 120 mg/kg groups at 24 h after administration. At the same time, AST returned to baseline, except in the 120 mg/kg group, where ALT peaked on day 7 and subsequently decreased but remained higher than baseline (Figures 1C,D). ALP, GGT, and TB peaked on day 7 and subsequently declined but remained above baseline by day 15 after administration in the 59 and 120 mg/kg groups (Figures 1E–G). GLU decreased at 24 h after administration, followed by recovery in the 59 and 120 mg/kg groups (Figure 1H). BUN and CREA increased at 24 h after administration, followed by recovery in the 59 mg/kg dose. In the 120 mg/kg group, BUN and CREA were elevated after administration, peaked on day 7, then gradually declined and returned to normal by day 15 (Figures 1I,J). CK and LDH increased at 24 h after administration, and then recovered to normal in the 59 and 120 mg/kg groups (Figures 1K,L). No significant differences were observed in the other dose groups. No obvious changes were observed in the control, 29, and 41 mg/kg groups. No obvious changes were observed in Ca^2+^, Na^+^, K^+^, and Cl^−^ across all doses (Supplementary Table S1). No obvious changes were observed in hematology (data not shown). These findings demonstrate that acute exposure to genipin at 59 and 120 mg/kg may cause dose-dependent, reversible hepatic and renal functional disturbances. Hematological and electrolyte parameters remain unaffected, suggesting that genipin did not induce obvious hematopoietic damage or severe electrolyte disturbances in the acute phase.

#### 3.1.4 Pathology

Gross necropsy: The 29, 41, and 59 mg/kg groups showed a smaller thymus and hyperemia of the intestinal mucosa. In the 120 mg/kg group, a smaller thymus, blue discoloration of the bilateral kidneys, gallbladder distension with bile accumulation, focal greenish discoloration of the hepatic surface (attributed to bile stasis), and duodenal mucosal hyperemia were identified. No obvious abnormalities were observed in the other organs (Table 1).

Histopathological examination: In the 59 and 120 mg/kg groups, centrilobular hepatocellular vacuolization and portal tract fibrous hyperplasia were observed. Kupffer cells phagocytosing brown granules in the portal tracts and hepatic sinusoids increased in the 120 mg/kg group. Thymic atrophy was observed in the 29, 41, 59, and 120 mg/kg groups. Epithelial vacuolization of the gallbladder was observed in the 29 and 59 mg/kg groups. In the 120 mg/kg dose, renal tubular basophilia, interstitial fibrosis, and mild interstitial lymphocyte infiltration were detected. Duodenal glandular dilation containing eosinophilic contents mixed with cellular debris was observed in the 29 and 120 mg/kg groups. Mild inflammatory cell aggregation was found in the cecal glandular ducts in the 59 mg/kg dose. Superficial autolysis of the cecal mucosa was identified in the 120 mg/kg dose; no obvious histopathological abnormalities were detected in the heart, spleen, or lung in any dose.

No obvious changes in electrocardiographic parameters and fecal occult blood were observed across all doses (data not shown).

### 3.2 28-Day subacute oral toxicity study in Beagle dogs

According to the results of the acute oral toxicity study, 27 mg/kg/day, a dose close to the lowest level that induced evident toxic effects in the acute study, was selected as the high dose for repeated administration to ensure clear toxicological responses in the subacute study. The middle and low doses were set at 9 and 3 mg/kg/day (Figure 2A).

**FIGURE 2:**
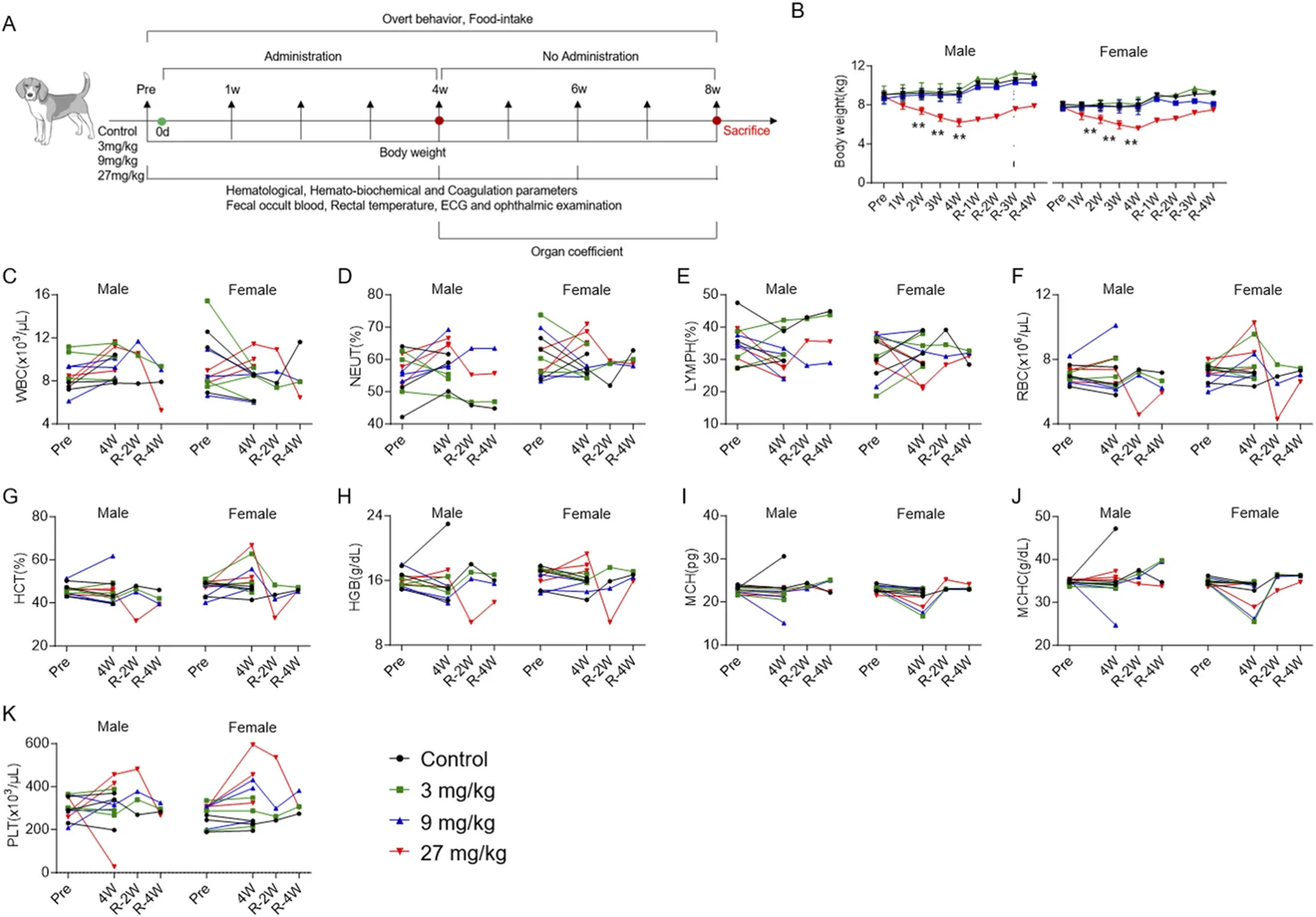
Body weight and hematology of Beagle dogs in the 28-day oral toxicity study of genipin. Data are presented as individual values, n = 3/group/sex, with lines depicting temporal changes in the respective indices for each dog. **(A)** Schematic diagram of 28-day oral toxicity study of genipin. **(B)** Time-course changes in body weight of genipin-treated dogs. n = 3. Data are presented as mean ± SD; *p* < 0.01 vs. control group. **(C–K)** Time-dependent changes in hematology, WBC **(C)**, NEUT% **(D)**, LYMPH% **(E)**, RBC **(F)**, HCT% **(G)**, HGB **(H)**, MCH **(I)**, MCHC **(J)**, and PLT **(K)**, following genipin administration and during the recovery period. n = 3/group/sex during the administration phase, n = 1/group/sex during the recovery phase.

#### 3.2.1 Clinical signs

Dogs (5/6) in the high-dose group vomited food after administration. No emesis was observed in the recovery period. Blue or bluish-black feces were observed in the high-dose (100%) and mid-dose (50%) groups throughout the administration period, with color returning to normal during the recovery period. No abnormalities were observed in the low-dose group. No mortality was observed in dogs.

#### 3.2.2 Body weight

Body weights of male and female dogs in the high-dose group were significantly decreased at weeks 2, 3, and 4 after administration compared with the control group (p < 0.01) (Figure 2B). After genipin withdrawal, body weights increased but remained lower than those of the control group at the end of the recovery period (Figure 2B). No significant differences were observed in the other groups during both the administration and recovery periods.

#### 3.2.3 Food intake

Food intake in the high-dose group decreased significantly, with almost complete anorexia at 1 week after administration. Radit test analysis showed that food intake in the high-dose group was significantly lower than in the control group (p < 0.01). To maintain the dogs’ vital signs, gavage of 100 g of food per dog was initiated on day 9 post-administration. Food intake began to recover and returned to normal by day 7 of the recovery period (Tables 3, 4). No significant differences were noted in the other groups during both the administration and recovery periods.

**TABLE 3:** Food intake during Subacute toxicity of genipin in Beagle dogs -Males.

Food intake	Control	3 mg/kg	9 mg/kg	27 mg/kg
No residue	68	63	78	9
Slight residue	12	14	6	7
Moderate residue	3	7	0	20
Complete residue	1	0	0	48
n	3	3	3	3
*p*	​	>0.05	>0.05	<0.01

n: number of Beagle dogs; *p*: compared with the control group.

**TABLE 4:** Food intake during Subacute toxicity of genipin in Beagle dogs -Females.

Food intake	Control	3 mg/kg	9 mg/kg	27 mg/kg
No residue	72	63	62	3
Slight residue	9	13	14	8
Moderate residue	2	8	8	8
Complete residue	1	0	0	65
n	3	3	3	3
*p*	​	>0.05	>0.05	<0.01

n: number of Beagle dogs; *p*: compared with the control group.

#### 3.2.4 Hematology and clinical chemistry

Hematology was assessed after 4 weeks of genipin administration and at 2 and 4 weeks of recovery. The results showed that in the high-dose group, WBC counts increased compared with pre-administration baseline (∼31% in males, ∼23% in females) and the control group (∼21% in males, ∼33% in females) (Figure 2C). During the recovery period, WBC counts remained elevated at 2 weeks, but decreased below pre-administration baseline by week 4 (Figure 2C). %NEUT increased after 4 weeks of administration, and the elevation in females was statistically significant compared with the control group (p < 0.05). %NEUT returned to normal at 2 weeks of recovery (Figure 2D). %LYMPH was decreased compared with pre-administration baseline (∼19% in males, ∼28% in females) and the control group (∼24% in males, ∼31% in females), but returned to baseline during the recovery period (Figure 2E). No significant differences were observed in %MONO, %BASO, and %EOS (Supplementary Tables S2, S3). These results suggest that high-dose genipin can induce systemic stress and mild inflammatory responses, which are reversible. RBC counts and HCT% were markedly elevated in one female dog in the high-dose group, with no significant differences observed in all other groups. During recovery, RBC counts, HCT%, and HGB in the high-dose group decreased at 2 weeks of recovery but returned to baseline by the end of the recovery period (Figures 2F–H). Although HGB in female dogs in the high-dose group was statistically significantly lower than in the control group (p < 0.01), the increase was 9.7% and not biologically meaningful. A reduction in MCH and MCHC was observed in the male mid-dose group, primarily attributed to a marked decrease (MCH: ∼31%, MCHC: ∼30%) in one dog. Individual instances of significant declines in MCH and MCHC were observed in a single dog across the female low-, mid-, and high-dose groups (Figures 2I,J). During recovery, MCH and MCHC levels in female dogs returned to baseline by 2 weeks. No significant differences were observed in MCV (Supplementary Tables S2, S3). PLT counts were elevated in the male high-dose group and in the female mid- and high-dose groups after 4 weeks of administration. During the recovery phase, PLT elevation persisted in the high-dose group at 2 weeks, but returned to baseline by the end of recovery (Figure 2K). These findings indicate that high-dose genipin induced hematological alterations, mainly attributed to systemic stress responses triggered by gastrointestinal irritation and hemoconcentration secondary to reduced food and water intake. At the 2-week recovery period, the high-dose group exhibited hematological changes consistent with nutritional deficiency-related anemia.

To evaluate the potential toxicity of genipin on the coagulation-fibrinolytic system, coagulation parameters were measured. The results showed that in the high-dose group, APTT and TT were significantly increased after 4 weeks of administration compared with the pre-administration baseline (APTT: ∼54% in males, ∼49% in females; TT: ∼34% in males, ∼27% in females) and the control group (APTT: ∼72% in males, ∼51% in females; TT: ∼36% in males, ∼34% in females). All indices returned to baseline by the end of recovery (Figures 3A, B). FIB was significantly increased compared with pre-administration baseline (∼53% in males, ∼71% in females) and the control group (∼20% in males, ∼50% in females). At the 2-week recovery period, FIB in the high-dose group continued to rise, increasing to ∼86% in males and ∼188% in females. By the end of recovery, FIB showed partial recovery but remained higher than the pre-administration baseline (∼62% in males, ∼52% in females) (Figure 3C). No significant PT abnormalities were observed in any group (Supplementary Tables S4, S5). These results suggest that high-dose genipin may directly inhibit the intrinsic coagulation pathway rather than causing insufficient coagulation factor synthesis after 4 weeks of administration, with a reversible tendency toward decreased coagulation function. Elevated FIB indicates systemic stress and inflammation, consistent with previous findings.

**FIGURE 3:**
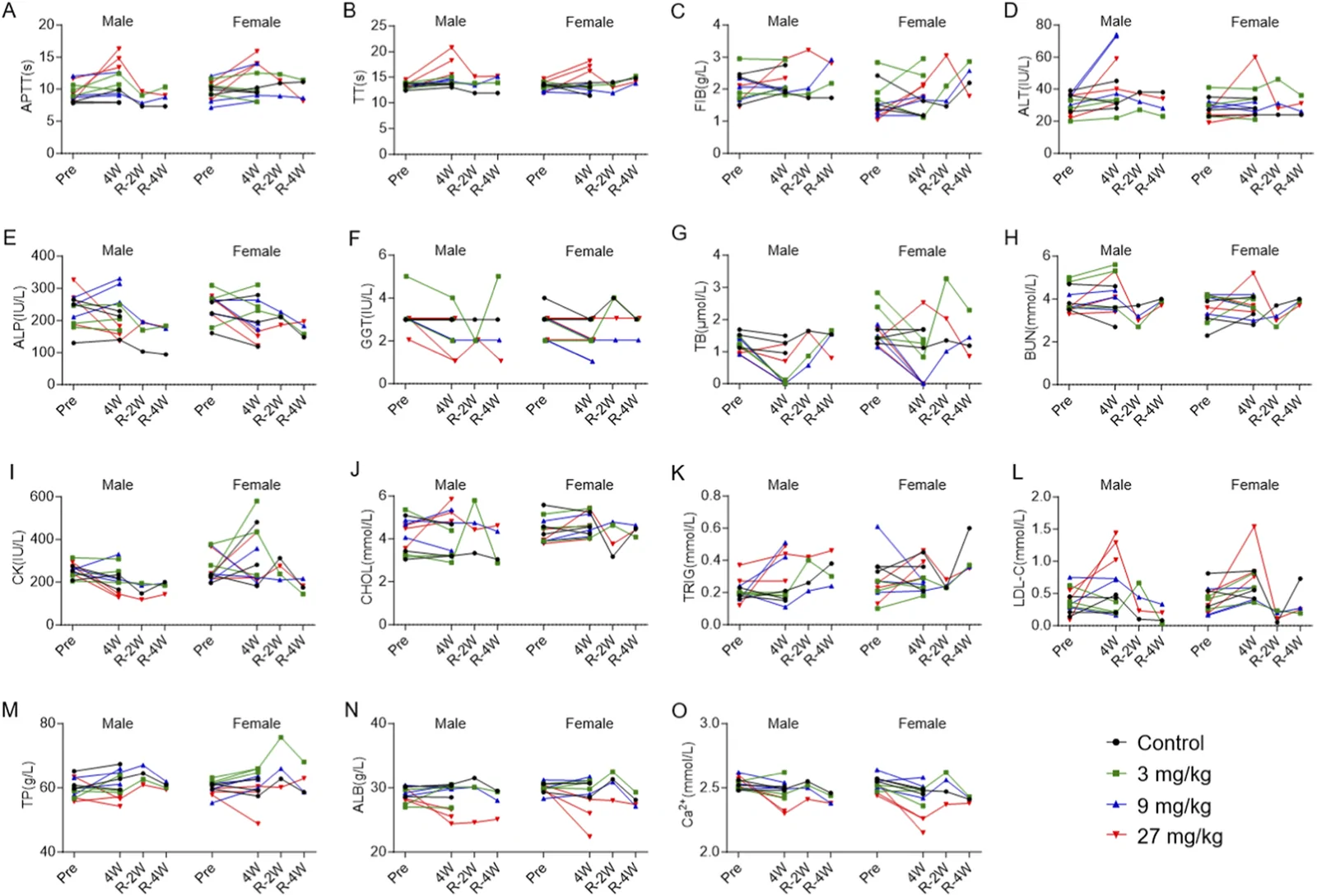
Coagulation and Hematochemical parameters of Beagle dogs in the 28-day oral toxicity study of genipin. Data are presented as individual values, n = 3/group/sex. **(A–C)** Time-dependent changes in coagulation parameters, APTT **(A)**, TT **(B)**, and FIB**(C)**, after genipin administration and during the recovery period. **(D–O)** Time-dependent changes in clinical chemistry, ALT **(D)**, ALP **(E)**, GGT **(F)**, TB **(G)**, BUN **(H)**, CK **(I)**, CHOL **(J)**, TRIG **(K)**, LDL-C **(L)**, TP **(M)**, ALB **(N)**, and Ca^2+^ **(O)**, after genipin administration and during the recovery period. n = 3/group/sex during the administration phase, n = 1/group/sex during the recovery phase.

To evaluate genipin’s potential effects on vital organ function, metabolism, and electrolyte balance, clinical chemistry was performed. After 4 weeks of administration, the results showed a marked elevation in ALT in one male and one female dog in the high-dose group, with levels returning to baseline after 2 weeks of recovery. In the male mid-dose group, ALT was significantly increased relative to both pre-administration baseline and the control group (Figure 3D). ALP was elevated by ∼23% and ∼55% in the male mid-dose group compared with pre-administration baseline and the control group, respectively. In both male and female high-dose groups, ALP was decreased relative to pre-administration baseline (∼39% in males, ∼42% in females) and decreased relative to the control group (∼18% in males, ∼26% in females). ALP levels subsequently showed partial recovery but remained lower than pre-administration baseline (Figure 3E). GGT was decreased in the mid- and high-dose group compared with pre-administration (mid-dose: ∼33%–43%; high-dose: ∼13%–37%) and the control group (mid-dose: ∼33%–56%; high-dose: ∼22%–44%). All groups showed partial recovery of GGT during the recovery period (Figure 3F; Supplementary Tables S6, S7). TB was markedly decreased in all dose groups relative to both pre-administration baseline (low-dose: ∼48–95%; mid-dose: 100%; high-dose: ∼34–41%) and the control group (low-dose: ∼23–49%; mid-dose: 100%; high-dose: ∼44–48%). By the end of the recovery period, TB was basically restored (Figure 3G). These changes may reflect reversible hepatic dysfunction and secondary hematopoietic effects. Compared with pre-administration, BUN was elevated in one high-dose dog of each sex (∼18% in males; ∼26% in females), with recovery observed by the end of the recovery period (Figure 3H). CK decreased by ∼50% and ∼34% in the male high-dose group compared with pre-administration and the control group, respectively. At the end of the recovery period, CK in the male high-dose group remained lower than pre-administration (Figure 3I). CHOL increased by ∼25% and ∼44% in the male high-dose group compared with pre-administration and the control group, respectively, and returned to normal during the recovery period (Figure 3J). TRIG was increased in the male mid- and high-dose groups compared with pre-administration (mid-dose: ∼73%; high-dose: ∼58%) and the control group (mid-dose: ∼100%; high-dose: ∼131%). In the female low- and high-dose groups, TRIG was also increased by ∼21% and ∼84%, respectively. These elevations persisted throughout the recovery period (Figure 3K). LDL was increased in the high-dose groups relative to pre-administration baseline (∼257% in males, ∼211% in females) and the control group (∼238% in males, ∼73% in females). LDL in the female low-dose group was elevated by ∼57% relative to pre-administration. During the recovery period, LDL returned to baseline (Figure 3L). TP in the male high-dose group was significantly decreased by ∼11% compared with the control group (p < 0.05; Figure 3M). ALB was reduced in both male and female high-dose groups relative to pre-administration (∼39% in males; ∼42% in females) and the control group (∼14% in males; ∼15% in females), with partial recovery observed by the end of the recovery period (Figure 3N). Ca^2+^ in the female high-dose group was significantly decreased by ∼10% compared with the control group (p < 0.05; Figure 3O). No significant abnormalities in AST, CREA, GLU, HDL, Na^+^, K^+^, and Cl^−^ were observed in all groups during the 4-week administration period and the recovery period (Supplementary Tables S6, S7).

#### 3.2.5 Urinalysis

To evaluate the potential effects of genipin on renal function and the urinary system in Beagle dogs, urine samples were collected and analyzed. The results showed that urine from the high-dose group exhibited blue-black discoloration after 4 weeks of administration. Dose-dependent increases in positivity rates were observed for UBG, BIL, and ERY in mid- and high-dose groups. During the recovery period, UBG and BIL returned to normal, whereas the female low-dose group showed persistent strong ERY positivity (Tables 5, 6). These results suggest that high-dose genipin may induce mild hepatobiliary metabolic alterations and transient irritation of the urinary tract mucosa.

**TABLE 5:** Urinalysis during Repeated-dose toxicity of genipin in Beagle dogs-end of Administration.

Group	Sex	Color	LEU	PRO	GLU	KET	UBG	BIL	ERY	NIT
Control	♂	Amber	-	+	-	+	+	+	-	-
	♂	Yellow	-	-	-	-	-	-	-	-
	♀	Brown	+	+	-	+	+	-	-	-
	♀	Amber	-	-	-	+	-	-	-	-
3 mg/kg	♂	Amber	-	+	-	+	++	+	-	-
	♂	Yellow	-	+	-	-	-	+	-	-
	♀	Amber	-	+	-	+	-	+	-	-
	♀	Amber	-	-	-	+	-	-	-	-
9 mg/kg	♂	Amber	-	-	-	-	+	+	-	-
	♂	Yellow	-	-	-	-	-	-	-	-
	♀	Amber	-	+	-	+	++	+	-	-
	♀	Brown	-	+	-	+	+	-	++	-
27 mg/kg	♂	Brown	+	+	-	+	+++	++	+	—
	♂	Others	-	+	-	+	++	+	+	-
	♀	Others	+	+	-	+	+++	+	++	-
	♀	Others	+	+	-	+	+++	+	+	-

**TABLE 6:** Urinalysis during Repeated-dose toxicity of genipin in Beagle dogs-end of Recovery.

Group	Sex	Color	LEU	PRO	GLU	KET	UBG	BIL	ERY	NIT
Control	♂	Amber	-	+	-	+	+	+	-	-
	♀	Yellow	-	-	-	-	-	-	-	-
3 mg/kg	♂	Yellow	-	-	-	-	-	+	-	-
	♀	Amber	-	+	-	+	+	+	+++	-
9 mg/kg	♂	Amber	-	+	-	-	+	+	+	-
	♀	—	—	—	—	—	—	—	—	—
27 mg/kg	♂	Amber	+	+	-	+	+	-	-	-
	♀	Yellow	-	-	-	-	-	-	-	-

“/”: urine not collected.

#### 3.2.6 Electrocardiography (ECG)

To evaluate the potential cardiotoxicity of genipin on cardiac electrophysiology, ECG was monitored after administration and during the recovery period. The results showed that the ST segment was significantly decreased in the male high-dose group compared with the control group (p < 0.01) during the 4-week administration period and the recovery period. QT interval was shortened in the female low-dose group compared with pre-administration (p < 0.05). Although PR interval and heart rate changed significantly compared with pre-administration, these alterations were not biologically relevant (Supplementary Tables S8, S9).

#### 3.2.7 Pathology

Gross necropsy: After 4 weeks of oral genipin administration in Beagle dogs, the liver, bilateral kidneys, salivary glands, and mesenteric lymph nodes of all animals in the high-dose group exhibited blue discoloration (6/6). One male and one female dog in the high-dose group exhibited multiple pinhead-sized white mucosal defects in the duodenum. In male dogs, the high-dose group showed small bilateral testes, epididymides, and prostates (2/3), gallbladder mucosal hyperemia (1/3), and pyloric gastric mucosa pallor (1/3); the mid-dose group had small prostates (1/3); and the low-dose group showed smaller bilateral testes and prostates (1/3). In female dogs, the high-dose group showed small uteri (2/3), a small thymus (1/3), and white plaques on the splenic surface (1/3). The mid-dose group showed blue discoloration of the liver and bilateral kidneys (1/3) and white plaques on the lung margins (1/3). In the low-dose group, one dog exhibited duodenal mucosa hyperemia (Supplementary Tables S10–S12). During the recovery period, high-dose group dogs showed mild blue discoloration of the liver, bilateral kidneys, and salivary glands (2/2) and a slightly smaller thymus (2/2). Male dogs in the high-dose group presented with small bilateral testes and epididymides, while mid-dose male dogs had multiple pinhead-sized white defects in the duodenal mucosa. Female dogs in the high-dose group had small uteri, bilateral ovaries, and one pinhead-sized white defect in the duodenal mucosa. Low-dose female dogs showed slightly smaller bilateral ovaries. No significant abnormalities were noted in the body surface or other visceral organs of the remaining dogs (Supplementary Tables S10–S12).

Organ indices: After 4 weeks of oral genipin administration, compared with the control group, the testicular index (low-dose: ∼28%; mid-dose: ∼14%; high-dose: ∼65%) and epididymal index (low-dose: ∼9%; mid-dose: ∼21%; high-dose: ∼45%) in all male groups were decreased in a dose-dependent manner. The thymic index was increased in all groups (low-dose: ∼60%; mid-dose: ∼21%; high-dose: ∼14%). The adrenal index was elevated in both mid- and high-dose groups (mid-dose: ∼31%; high-dose: ∼81%) in a dose-dependent manner. In the high-dose group, the hepatic index (∼41%), pulmonary index (∼21%), renal index (∼30%), cerebral index (∼27%), and pituitary index (∼21%) were increased. In the female high-dose group, the uterine index (∼61%) and thymic index (∼39%) decreased, whereas the hepatic index (∼27%), splenic index (∼43%), and cerebral index (∼36%) increased. The adrenal index increased in a dose-dependent manner (low-dose: ∼21%; mid-dose: ∼29%; high-dose: ∼82%). The ovarian index was elevated across all groups (low-dose: ∼43%; mid-dose: ∼48%; high-dose: ∼38%). The splenic index (∼31%) and uterine index (∼138%) were increased. In the mid-dose group, the thymic index increased (∼75%) (Supplementary Tables S13–S16).

Histopathological examination: After 4 weeks of oral genipin administration, the high-dose group exhibited toxic effects on the gonads, bone marrow, thymus, and liver, and an irritant effect on the gastric mucosa. The low- and mid-dose groups also induced toxic effects on the male gonads. After a 4-week recovery period following drug withdrawal, the toxic effects on the bone marrow and the irritant effect on the gastric mucosa were basically recovered; the toxic effects on the liver, thymus, and gonads were partially ameliorated (Figures 4A,B; Supplementary Tables S10–S12).

**FIGURE 4:**
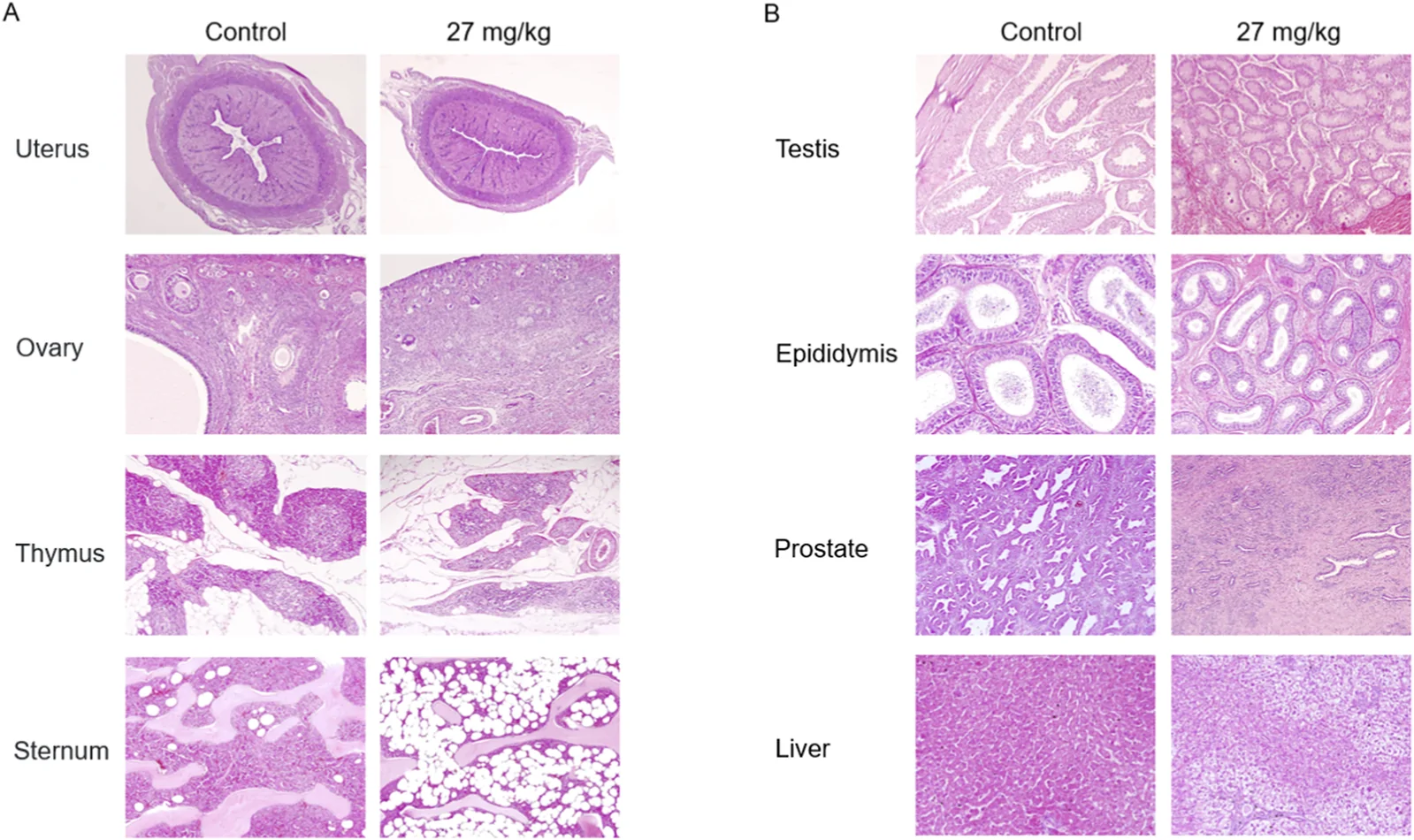
Histopathology of organs in female **(A)** and male **(B)** dogs after 28-day genipin treatment. H&E staining; Uterus: 40x; Epididymis and Liver: 200x; other organs: 100x.

Fecal occult blood was negative in all groups. No significant abnormalities in body temperature or ophthalmological examination results were observed in any group during the 4-week genipin administration period and the recovery period.

## 4 Discussion

Genipin, a natural iridoid derivative, has garnered extensive attention in food science, biomedicine, and material engineering owing to its unique biological activities and crosslinking properties. However, the scarcity of comprehensive safety data and inconsistencies in existing toxicological findings have raised legitimate concerns regarding its widespread application. Therefore, our study systematically evaluated the acute and subchronic toxic effects of genipin in Beagle dogs—a non-rodent model with metabolic characteristics highly analogous to humans—and established critical safe exposure thresholds. We conducted a single-dose acute oral toxicity study and a 28-day repeated oral toxicity study, followed by a 4-week recovery period, to comprehensively assess toxicity and its reversibility.

Our findings identified the approximate lethal dose (ALD) of genipin following a single oral administration in Beagle dogs as 120–140 mg/kg, a substantially higher concentration than the doses encountered in routine industrial or biomedical applications. In the 28-day repeated-dose study, high-dose genipin (27 mg/kg/day) induced multi-organ toxicity, but most injuries exhibited reversibility following drug withdrawal. In contrast, the toxic effects of mid- and low-dose genipin (9 and 3 mg/kg/day) were mild, limited, and fully reversible. This dose-dependent toxicity is consistent with the findings from our prior repeated-dose toxicity study of genipin in rats. Previous studies also show that genipin induces overt hepatotoxicity in mice/rats at high doses, with significant amelioration of hepatic lesions following drug withdrawal (Li et al., 2019; Liu et al., 2025; Yamano et al., 1990; Wang et al., 2023). Notably, the no-observed-adverse-effect level (NOAEL) of genipin in Beagle dogs (below 3 mg/kg/day) is significantly higher than the typical exposure levels in food additives (Castle et al., 2025) (e.g., gardenia blue pigment (China, 2025), a genipin-derived food colorant) and biomedical formulations, underscoring its relative safety under routine applications.

Gastrointestinal irritation is the core toxic manifestation of genipin, directly eliciting emesis, reduced food intake, and body weight loss in high-dose Beagle dogs, consistent with the mucosal erosion and regenerative lesions identified histopathologically. Our previous study also found that genipin exerted certain irritant effects on the gastric mucosa of rats. Importantly, such irritant injuries are nearly fully reversible following drug withdrawal, a critical consideration for clinical safety evaluation. In contrast, Lee et al. and Sohn et al. reported that genipin dose-dependently mitigates gastric injury in rats via gastric acid neutralization, inhibition of H^+^/K^+^-ATPase activity, and upregulation of the mucosal protective factor prostaglandin E_2_ in gastric cells (Lee et al., 2009; Sohn et al., 2017). Zhao et al. further confirmed that genipin alleviates acute gastric injury in mice by suppressing NLRP3 inflammasome activation (Zhao et al., 2020). Notably, our study employed Beagle dogs, which are highly sensitive to hepatotoxic and gastrointestinal toxicants. Combined with previous investigations into genipin’s gastroprotective properties, our results underscore the imperative of dose optimization and context-specific risk assessment for genipin’s applications in food science, biomedicine, and materials engineering. Future research should prioritize defining genipin’s dose-response relationship in human gastrointestinal models and elucidating its protective mechanisms under clinically relevant conditions to reconcile its dual effects, maximize therapeutic potential while minimize toxicity risks.

The present study identified key target organs for genipin toxicity, with distinct profiles of reversibility. Genipin, as the reactive metabolite of geniposide, is responsible for geniposide-induced hepatotoxicity (Zhao et al., 2023). In our study, we also found that the high dose of genipin induced reversible functional hepatic perturbations, and all hepatic functional abnormalities were significantly ameliorated or completely restored by the end of the recovery period. This reversibility, coupled with the absence of severe hepatocellular necrosis or structural damage, aligns with the finding that genipin-induced hepatic injury is predominantly functional rather than organic. This feature distinguishes it from severe hepatotoxicants. Wang et al. found that genipin-induced liver injury in mice is associated with the dysregulation of the UDP-glucuronosyltransferase (UGT) system and cytochrome P450 enzyme activity (Wang et al., 2023). The high dose of genipin also induced reversible bone marrow hematopoietic suppression that subsequently led to anemia. Corresponding coagulation abnormalities suggested that genipin or its metabolites may directly inhibit the intrinsic coagulation pathway. This study also revealed the key targets of potential irreversible damage induced by genipin. The high dose significantly reduced the relative organ weights of the testes, epididymides, and thymus, accompanied by corresponding histopathological lesions. By the end of the recovery period, the relative organ coefficients and microstructures of these organs had not returned to normal, indicating that genipin may exert persistent effects on the reproductive and immune organs.

Previous Studies reported that genipin mitigates fertility and spermatogenesis impairment induced by circadian rhythm disruption and restores testosterone, dihydrotestosterone, and androstenedione levels in mice (Xu et al., 2020). Our previous study also failed to detect reproductive toxicity of genipin in rats. Such species differences are common in toxicological research and are primarily attributed to interspecies variations in the composition and expression levels of metabolic enzyme subtypes, including UDP-glucuronosyltransferases (UGTs) and cytochrome P450 enzymes. Specifically, Beagle dogs may exhibit higher metabolic activation efficiency of genipin than rats, facilitating the accumulation of toxic metabolites in target organs; additionally, dogs are inherently more sensitive to immunotoxicants. These results highlight the necessity of integrating toxicological data from both non-rodent (e.g., dogs) and rodent (e.g., rats) models for a comprehensive safety assessment of genipin.

The blue pigmentation observed in the feces, urine, and visceral organs of genipin-treated dogs was confirmed to be a physical color-development process resulting from the conjugation of genipin with amino acids to form gardenia blue pigment, rather than a pathological alteration associated with toxicity (Tsutsumiuchi et al., 2021).

This study has several limitations that should be acknowledged. First, the small number of Beagle dogs in the 28-day repeated-dose study may have increased the influence of individual biological variation. Additional independent replicated animal experiments are required to corroborate the toxicological outcomes in Beagle dogs during the recovery phase. Second, the lack of toxicokinetic (TK) evaluation precluded establishing a definitive dose–exposure–toxicity relationship. In addition, the precise toxic mechanisms underlying the observed hepatic, thymic, and gonadal injury were not fully elucidated without targeted mechanistic studies.

Collectively, genipin toxicity may be attributed to the covalent binding of its active metabolites to biological macromolecules. Gastrointestinal irritation was the primary initiating event, and the systemic stress, dehydration, and insufficient nutrient intake exacerbated subsequent adverse reactions (e.g., hemoconcentration and elevated blood urea nitrogen). As the major metabolic organ, the liver endured the first-pass effect and the metabolic burden of genipin, but hepatic damage was predominantly functional and reversible. However, potential persistent damage to the gonads and thymus warrants serious attention and may become a key limiting factor in assessing its long-term safety. These findings provide crucial dose-response data and safety-threshold evidence for the risk assessment of genipin applications in the food and biomedical fields.

## 5 Conclusion

The present study clarified the dose-toxicity relationship, major target organs, and toxic reversibility of genipin following oral exposure in non-rodent species. These findings provide robust preclinical toxicological evidence to support rational dosage optimization, risk assessment, and the safe utilization of genipin. From an ethnopharmacological perspective, these findings highlight the need for strict dose regulation and safety monitoring for the oral use of genipin-related traditional preparations, complementing and optimizing the modern safety evaluation system of gardenia-based ethnomedicine.

## Data Availability

The original contributions presented in the study are included in the article/Supplementary Material, further inquiries can be directed to the corresponding author.

## Glossary

GLP: Good Laboratory Practice
NMPA: National Medical Products Administration
OECD: Organization for Economic Cooperation and Development
MLD: Minimum lethal dose
MNLD: Maximum non-lethal dose
NOAEL: No-observed-adverse-effect level ()
ECG: Electrocardiogram
RBC: Red blood cell
HGB: Hemoglobin
WBC: White blood cell
HCT: Hematocrit
MCV: Mean corpuscular volume
MCH: Mean corpuscular hemoglobin
MCHC: Mean corpuscular hemoglobin concentration
**P**LT: Platelet count
NEUT: Neutrophil
LYMPH: Lymphocyte
MONO: Monocyte
EOS: Eosinophil
BASO: Basophil
PT: Prothrombin time
APTT: Activated partial thromboplastin time
TT: Thrombin time
FIB: Fibrinogen
AST: Aspartate aminotransferase
ALT: Alanine aminotransferase
ALP: Alkaline phosphatase
BUN: Blood urea nitrogen
TB: Total bilirubin
CK: Creatine kinase
GGT: γ-Glutamyl transferase
LDH: Lactate dehydrogenase
TP: Total protein
CHOL: Total cholesterol
LDL: Low-density lipoprotein
HDL: High-density lipoprotein
TG: Triglycerides
CREA: Creatinine
SG: Specific gravity
PRO: Protein
KET: Ketone bodies
LEU: Leukocytes
ERY: Erythrocyte
UBG: Urobilinogen
BIL: Bilirubin
NIT: Nitrite
