# Supplementary material for: Safety evaluation of genipin in Beagle dogs: acute and 28-day repeated oral toxicity studies
Source: Front Pharmacol. 2026 Oct 30;17:1924890. doi: 10.3389/fphar.2026.1924890 (PMC13759790; doi:10.3389/fphar.2026.1924890)
Supplement: Supplementary file 1 [file Supplementaryfile1.docx]

Supplementary Material

# 1 Supplementary Tables

**Table S1.** Clinical chemistry parameters in Beagle dogs from the acute oral toxicity study of genipin

| **Item**  **Gruop** | | **ALT (IU/L)** | **AST (IU/L)** | **TBIL (μmol/ L)** | | **ALP**  **(IU/L)** | **GGT**  **(IU/L)** | **GLU**  **(mmol/ L)** | **BUN**  **(mmol/ L)** | **CREA**  **(µmol/ L)** | **Ca**  **(mmol/ L)** | **CK**  **(IU/L)** | **LDH**  **(IU/L)** | **Na^+^**  **(mmol/ L)** | **K^+^**  **(mmol/ L)** | **Cl^-^**  **(mmol/ L)** |
| --- | --- | --- | --- | --- | --- | --- | --- | --- | --- | --- | --- | --- | --- | --- | --- | --- |
| Control | Pre | 26 | 29 | 1.83 | 125 | | 4 | 3.98 | 5.3 | 63 | 2.62 | 207 | 138 | 150.0 | 4.92 | 115.3 |
|  | 24h | 70 | 92 | 0.97 | 120 | | 4 | 4.19 | 5.6 | 59 | 2.62 | 146 | 86 | 140.3 | 5.13 | 109.8 |
|  | 7day | 25 | 29 | 1.08 | 120 | | 4 | 3.43 | 6.6 | 62 | 2.66 | 205 | 155 | 150.2 | 5.45 | 113.8 |
|  | 10day | 26 | 30 | 1.26 | 120 | | 4 | 3.98 | 4.6 | 50 | 2.62 | 219 | 187 | 151.3 | 5.41 | 115.1 |
|  | 15day | 28 | 31 | 1.46 | 110 | | 4 | 3.83 | 6.4 | 60 | 2.66 | 258 | 111 | 150.3 | 5.37 | 114.7 |
| 29mg/kg | Pre | 31 | 31 | 2.07 | 181 | | 3 | 5.04 | 3.8 | 58 | 2.53 | 265 | 154 | 150.0 | 5.41 | 116.9 |
|  | 24h | 53 | 36 | -0.98 | 238 | | 4 | 4.24 | 6.2 | 52 | 2.58 | 225 | 173 | 153.7 | 5.40 | 119.9 |
|  | 7day | 39 | 28 | 0.52 | 222 | | 3 | 5.7 | 5.2 | 48 | 2.69 | 233 | 271 | 156.3 | 5.68 | 124.3 |
|  | 10day | 40 | 33 | 0.81 | 219 | | 4 | 5.38 | 4.3 | 51 | 2.7 | 237 | 138 | 153.3 | 5.32 | 120.2 |
|  | 15day | 36 | 33 | 0.7 | 181 | | 4 | 4.88 | 3.5 | 46 | 2.18 | 208 | 101 | 149.2 | 5.17 | 114.2 |
| 41mg/kg | Pre | 52 | 31 | 1.72 | 223 | | 3 | 4.54 | 4.8 | 65 | 2.67 | 178 | 110 | 149.6 | 4.87 | 115.8 |
|  | 24h | 120 | 80 | -0.82 | 296 | | 4 | 4.38 | 5.9 | 63 | 2.63 | 184 | 168 | 134.9 | 5.01 | 103.7 |
|  | 7day | 71 | 36 | 1.54 | 205 | | 4 | 4.53 | 5.4 | 58 | 2.62 | 178 | 209 | 149.4 | 4.78 | 113.1 |
|  | 10day | 62 | 36 | 1.56 | 206 | | 4 | 4.52 | 6.1 | 50 | 2.65 | 181 | 203 | 150.8 | 4.85 | 113.7 |
|  | 15day | 46 | 32 | 1.37 | 182 | | 4 | 4.17 | 5.1 | 58 | 2.62 | 189 | 210 | 149.2 | 4.87 | 113.5 |
| 59mg/kg | Pre | 22 | 33 | 1.94 | 178 | | 4 | 3.95 | 5.3 | 60 | 2.51 | 268 | 181 | 150.2 | 4.71 | 116.4 |
|  | 24h | 1796 | 5476 | 0.45 | 363 | | 9 | 2.7 | 9 | 72 | 2.5 | 572 | 1015 | 134.8 | 4.98 | 105.3 |
|  | 7day | 1237 | 189 | 6.03 | 1291 | | 20 | 3.49 | 3.6 | 61 | 2.52 | 217 | 185 | 150.1 | 4.66 | 115.4 |
|  | 10day | 517 | 97 | 2.43 | 618 | | 11 | 4.05 | 4.7 | 44 | 2.7 | 186 | 171 | 151.6 | 5.12 | 114.5 |
|  | 15day | 146 | 50 | 1.73 | 312 | | 8 | 4.04 | 8 | 52 | 0.09 | 12 | 205 | 150.8 | 5.22 | 116.3 |
| 84mg/kg | Pre | 25 | 29 | 1.28 | 192 | | 3 | 4.48 | 3.8 | 55 | 2.50 | 149 | 96 | 149.3 | 4.33 | 116.5 |
|  | 24h | 40 | 55 | 0.35 | 202 | | 3 | 4.07 | 4.1 | 56 | 2.57 | 134 | 122 | 136.2 | 5.00 | 104.9 |
|  | 7day | 27 | 33 | 1.01 | 192 | | 2 | 3.98 | 4.8 | 57 | 2.6 | 138 | 128 | 149.3 | 4.12 | 114.7 |
|  | 10day | 25 | 33 | 1.76 | 192 | | 3 | 3.55 | 4.5 | 51 | 2.64 | 141 | 198 | 150.8 | 4.22 | 114.7 |
|  | 15day | 29 | 35 | 1.65 | 206 | | 3 | 3.79 | 3.8 | 53 | 2.71 | 150 | 148 | 148.5 | 4.76 | 114.3 |
| 120mg/kg | Pre | 28 | 28 | 1.87 | 207 | | 3 | 4.39 | 4.4 | 68 | 2.58 | 210 | 104 | 149.2 | 4.60 | 115.5 |
|  | 24h | 1328 | 5439 | 10.91 | 551 | | 7 | 3.07 | 9.3 | 88 | 2.6 | 816 | 1333 | 140.2 | 5.05 | 110.2 |
|  | 7day | 2273 | 601 | 226.48 | 5677 | | 45 | 4.58 | 11.9 | 125 | 2.47 | 276 | 116 | 142 | 3.74 | 106.9 |
|  | 10day | 1502 | 335 | 35.51 | 4854 | | 36 | 4.3 | 6.8 | 78 | 2.6 | 244 | 104 | 149.8 | 4.65 | 113.8 |
|  | 15day | 666 | 98 | 14.39 | 2131 | | 22 | 3.67 | 5 | 64 | 2.68 | 149 | 104 | 149.8 | 4.90 | 114.2 |
| 140mg/kg | Pre | 69 | 44 | 2.01 | 230 | | 3 | 4.42 | 5.8 | 52 | 2.60 | 338 | 172 | 151.0 | 4.96 | 118.2 |
|  | 24h | 290 | 296 | -0.44 | 545 | | 4 | 3.37 | 7.7 | 46 | 2.5 | 285 | 366 | 147.2 | 4.85 | 112.1 |
|  | 7day | 653 | 70 | 1.05 | 622 | | 5 | 5.25 | 5.3 | 39 | 2.64 | 186 | 71 | 152.6 | 5.38 | 119.5 |
|  | 10day | 264 | 54 | 0.09 | 449 | | 5 | 5.36 | 4.4 | 45 | 2.74 | 267 | 101 | 152.6 | 4.66 | 119.2 |
|  | 15day | 93 | 35 | 0.09 | 299 | | 4 | 5.53 | 4.8 | 37 | 2.63 | 159 | 125 | 146.8 | 4.52 | 113.6 |

n=1

**Table S2**. Hematological parameters in Beagle dogs from repeated oral administration of genipin-Males

| **Item** | **Group** | **Pre-Administration** | **Administration** | **Recovery-2W** | **Recovery-4W** |
| --- | --- | --- | --- | --- | --- |
| WBC  （×10^3^/μL） | Control | 7.52±0.33 | 8.75 ± 1.47 | 7.76 | 7.90 |
|  | 3 mg/kg | 10.02±1.58 | 9.95 ± 1.75 | 10.37 | 9.35 |
|  | 9 mg/kg | 8.27±1.86 | 9.20 ± 0.90 | 11.69 | 9.03 |
|  | 27 mg/kg | 8.07 ±0.40 | 10.59 ± 1.44 | 10.55 | 5.24 |
| RBC  （×10^6^/μL） | Control | 6.97±0.67 | 6.59 ± 0.87 | 7.36 | 7.18 |
|  | 3 mg/kg | 6.98±0.21 | 7.11 ± 0.87 | 7.21 | 6.66 |
|  | 9 mg/kg | 7.23±0.87 | 7.48 ± 2.27 | 7.01 | 6.23 |
|  | 27 mg/kg | 7.11±0.43 | 7.33 ± 0.79 | 4.57 | 5.94 |
| HGB  （g/dL） | Control | 16.5±1.5 | 17.2 ± 5.1 | 18.0 | 16.0 |
|  | 3 mg/kg | 15.7±0.5 | 15.4 ± 1.0 | 17.0 | 16.7 |
|  | 9 mg/kg | 16.1±1.7 | 14.1 ± 1.1 | 16.2 | 15.6 |
|  | 27 mg/kg | 16.0±0.5 | 16.3 ± 1.1 | 10.8 | 13.3 |
| HCT  (％) | Control | 46.8±3.7 | 43.8 ± 4.6 | 47.9 | 46.0 |
|  | 3 mg/kg | 45.8±1.2 | 45.3 ± 3.7 | 46.4 | 42.0 |
|  | 9 mg/kg | 46.3±4.4 | 47.2 ± 12.6 | 45.0 | 39.5 |
|  | 27 mg/kg | 45.5±1.6 | 45.2 ± 1.7 | 31.6 | 39.4 |
| MCV  (fL) | Control | 67.3±1.4 | 66.7 ± 1.7 | 65.0 | 64.0 |
|  | 3 mg/kg | 65.7±1.6 | 63.9 ± 2.6 | 64.4 | 63.1 |
|  | 9 mg/kg | 64.2±1.6 | 63.5 ± 2.2 | 64.2 | 63.4 |
|  | 27 mg/kg | 64.2±1.8 | 61.9 ± 4.4 | 69.1 | 66.4 |
| MCH  (pg) | Control | 23.6±0.4 | 25.7 ± 4.2 | 24.4 | 22.2 |
|  | 3 mg/kg | 22.6±0.9 | 21.7 ± 1.2 | 23.6 | 25.1 |
|  | 9 mg/kg | 22.3±0.4 | 19.6 ± 3.9 | 23.0 | 25.0 |
|  | 27 mg/kg | 22.5±0.9 | 22.3 ± 1.0 | 23.7 | 22.4 |
| MCHC  (g/dL) | Control | 35.1±0.4 | 38.7 ± 7.4 | 37.5 | 34.7 |
|  | 3 mg/kg | 34.3±0.6 | 33.9 ± 0.6 | 36.7 | 39.8 |
|  | 9 mg/kg | 34.8±0.4 | 30.7 ± 5.2 | 35.9 | 39.5 |
|  | 27 mg/kg | 35.1±0.4 | 36.1 ± 1.1 | 34.3 | 33.8 |
| PLT  （×10^3^/μL） | Control | 291±62 | 302 ± 92 | 269 | 283 |
|  | 3 mg/kg | 322±38 | 315 ± 64 | 339 | 296 |
|  | 9 mg/kg | 287±79 | 316 ± 22 | 377 | 324 |
|  | 27 mg/kg | 297 ±48 | 299 ± 237 | 481 | 267 |
| RETIC  (％) | Control | 1.0±0.2 | 0.4 ± 0.2 | 0.8 | 0.7 |
|  | 3 mg/kg | 0.9±0.0 | 0.6 ± 0.3 | 0.6 | 0.5 |
|  | 9 mg/kg | 1.1±0.3 | 0.4 ± 0.2 | 1.5 | 1.6 |
|  | 27 mg/kg | 1.1±0.3 | 0.2 ± 0.2 | 9.3 | 1.6 |
| NEUT  (％) | Control | 52.5±11.0 | 56.9 ± 6.0 | 45.8 | 44.8 |
|  | 3 mg/kg | 57.5±6.6 | 52.6 ± 3.6 | 46.8 | 46.9 |
|  | 9 mg/kg | 55.0±1.6 | 61.7 ± 6.5 | 63.4 | 63.4 |
|  | 27 mg/kg | 57.3±4.7 | 65.2 ± 1.2 | 55.2 | 55.6 |
| LYMPH  (％) | Control | 36.8±10.2 | 32.6 ± 5.3 | 43.0 | 44.9 |
|  | 3 mg/kg | 32.2±5.9 | 37.7 ± 5.5 | 42.6 | 43.8 |
|  | 9 mg/kg | 35.6±1.7 | 29.6 ± 5.0 | 28.1 | 28.9 |
|  | 27 mg/kg | 34.6±4.7 | 26.3 ± 2.0 | 35.7 | 35.5 |
| MONO  (％) | Control | 6.5±2.2 | 6.3 ± 1.6 | 4.3 | 5.3 |
|  | 3 mg/kg | 6.9±2.8 | 6.3 ± 3.3 | 4.2 | 4.1 |
|  | 9 mg/kg | 5.3±1.2 | 5.4 ± 1.1 | 5.0 | 4.2 |
|  | 27 mg/kg | 4.5±0.8 | 6.8 ± 1.6 | 6.6 | 5.9 |
| EOS  (％) | Control | 3.0±0.9 | 3.3 ± 1.2 | 5.4 | 3.8 |
|  | 3 mg/kg | 2.6±1.3 | 2.7 ± 1.1 | 5.3 | 4.1 |
|  | 9 mg/kg | 2.4±0.4 | 2.4 ± 0.9 | 2.7 | 2.9 |
|  | 27 mg/kg | 2.6±1.1 | 1.2 ± 0.8 | 1.8 | 1.8 |
| BASO  (％) | Control | 0.9±0.5 | 0.7 ± 0.2 | 1.3 | 0.9 |
|  | 3 mg/kg | 0.6±0.1 | 0.6 ± 0.1 | 1.0 | 1.0 |
|  | 9 mg/kg | 1.5±0.7 | 0.7 ± 0.1 | 0.8 | 0.5 |
|  | 27 mg/kg | 0.8±0.2 | 0.5 ± 0.2 | 0.5 | 1.1 |

1. Pre-Administration: n=3; Administration: n=3; Recovery-2W: n=1; Recovery-4W: n=1;

2. Compared with the control group: **p*<0.05, ***p*<0.01.

**Table S3**. Hematological parameters in Beagle dogs from the repeated oral administration of genipin-Females

| **Item** | **Group** | **Pre-Administration** | **Administration** | **Recovery-2W** | **Recovery-4W** |
| --- | --- | --- | --- | --- | --- |
| WBC  （×10^3^/μL） | Control | 10.19±2.94 | 7.75 ± 1.40 | 7.79 | 11.62 |
|  | 3 mg/kg | 10.28±4.47 | 7.93 ± 1.64 | 7.39 | 7.93 |
|  | 9 mg/kg | 8.65±2.16 | 7.77 ± 1.52 | 8.87 | 7.98 |
|  | 27 mg/kg | 8.38±0.57 | 10.27 ± 1.05 | 10.9 | 6.45 |
| RBC  （×10^6^/μL） | Control | 7.15±0.55 | 6.88 ± 0.48 | 6.94 | 7.3 |
|  | 3 mg/kg | 7.54±0.15 | 7.97 ± 1.43 | 7.68 | 7.45 |
|  | 9 mg/kg | 6.49±0.54 | 7.40 ± 0.81 | 6.50 | 7.04 |
|  | 27 mg/kg | 7.38±0.55 | 8.72 ± 1.43 | 4.30 | 6.62 |
| HGB  （g/dL） | Control | 16.6±1.6 | 15.3 ± 1.5 | 15.9 | 16.7 |
|  | 3 mg/kg | 17.4±0.2 | 16.2 ± 0.6 | 17.6 | 17.1 |
|  | 9 mg/kg | 15.3±1.2 | 15.4 ± 0.7 | 15.0 | 16.4 |
|  | 27 mg/kg | 16.5±0.7 | 18.1 ± 1.1** | 10.8 | 15.9 |
| HCT  (％) | Control | 47.1±3.7 | 45.1 ± 3.3 | 43.7 | 45.9 |
|  | 3 mg/kg | 50.2±1.2 | 52.3 ± 9.4 | 48.3 | 47.2 |
|  | 9 mg/kg | 43.8±4.2 | 49.5 ± 5.4 | 41.7 | 45.2 |
|  | 27 mg/kg | 48.1±1.4 | 56.1 ± 9.3 | 33.0 | 45.9 |
| MCV  (fL) | Control | 65.9±1.2 | 65.6 ± 1.2 | 63.0 | 62.9 |
|  | 3 mg/kg | 66.6±0.7 | 65.6 ± 0.5 | 62.9 | 63.3 |
|  | 9 mg/kg | 67.3± 0.8 | 66.9 ± 0.6 | 64.1 | 64.2 |
|  | 27 mg/kg | 65.4±2.8 | 64.4 ± 2.6 | 76.8 | 69.4 |
| MCH  (pg) | Control | 23.1±1.0 | 22.1 ± 0.8 | 22.9 | 22.9 |
|  | 3 mg/kg | 23.1±0.3 | 20.7 ± 3.5 | 23.0 | 23.0 |
|  | 9 mg/kg | 23.6±0.4 | 21.1 ± 3.1 | 23.1 | 23.2 |
|  | 27 mg/kg | 22.4±1.0 | 21.0 ± 2.1 | 25.2 | 24.1 |
| MCHC  (g/dL) | Control | 35.1±1.0 | 33.8 ± 0.9 | 36.3 | 36.3 |
|  | 3 mg/kg | 34.7±0.5 | 31.6 ± 5.3 | 36.5 | 36.3 |
|  | 9 mg/kg | 35.0±0.7 | 31.5 ± 4.6 | 36.0 | 36.2 |
|  | 27 mg/kg | 34.3±0.6 | 32.7 ± 3.3 | 32.7 | 34.7 |
| PLT  （×10^3^/μL） | Control | 234±40 | 218 ± 32 | 243 | 274 |
|  | 3 mg/kg | 272±72 | 283 ± 67 | 261 | 307 |
|  | 9 mg/kg | 271±60 | 355 ± 101 | 299 | 381 |
|  | 27 mg/kg | 304±5 | 458 ± 136 | 536 | 303 |
| RETIC  (％) | Control | 1.2±0.2 | 0.7 ± 0.2 | 1.6 | 1.1 |
|  | 3 mg/kg | 1.1±0.3 | 0.5 ± 0.2 | 1.1 | 1.0 |
|  | 9 mg/kg | 0.9± 0.1 | 0.4 ± 0.3 | 0.6 | 0.4 |
|  | 27 mg/kg | 1.2±0.2 | 0.4 ± 0.2 | 12.1 | 1.8 |
| NEUT  (％) | Control | 61.1±6.6 | 58.1 ± 3.2 | 51.9 | 62.8 |
|  | 3 mg/kg | 63.4±9.2 | 58.3 ± 5.7 | 58.7 | 60.0 |
|  | 9 mg/kg | 59.3±9.1 | 56.6 ± 1.9 | 58.8 | 57.9 |
|  | 27 mg/kg | 58.1±4.4 | 68.2 ± 2.9* | 59.5 | 58.6 |
| LYMPH  (％) | Control | 30.4±4.9 | 33.1 ± 5.3 | 39.1 | 28.4 |
|  | 3 mg/kg | 28.7±9.2 | 33.3 ± 5.1 | 34.5 | 32.6 |
|  | 9 mg/kg | 32.2±9.2 | 34.5 ± 3.9 | 30.9 | 32.0 |
|  | 27 mg/kg | 34.4±4.8 | 23.7 ± 4.4 | 28.3 | 31.0 |
| MONO  (％) | Control | 5.4±0.5 | 5.3 ± 2.1 | 4.6 | 4.7 |
|  | 3 mg/kg | 5.2± 2.0 | 5.1 ± 0.8 | 3.0 | 3.7 |
|  | 9 mg/kg | 5.6±1.0 | 5.2 ± 1.9 | 6.0 | 6.3 |
|  | 27 mg/kg | 3.4±1.1 | 6.2 ± 2.0 | 7.8 | 5.0 |
| EOS  (％) | Control | 2.4±1.5 | 2.8 ± 1.5 | 3.7 | 3.5 |
|  | 3 mg/kg | 1.7±1.4 | 2.2 ± 1.6 | 2.7 | 2.5 |
|  | 9 mg/kg | 1.9±0.3 | 2.6 ± 1.1 | 3.0 | 2.4 |
|  | 27 mg/kg | 3.0±0.4 | 1.1 ± 0.6 | 3.8 | 4.6 |
| BASO  (％) | Control | 0.5±0.2 | 0.5 ± 0.1 | 0.7 | 0.4 |
|  | 3 mg/kg | 0.7±0.3 | 0.9 ± 0.5 | 1.0 | 1.0 |
|  | 9 mg/kg | 0.7±0.3 | 0.8 ± 0.4 | 1.2 | 1.3 |
|  | 27 mg/kg | 0.9±0.2 | 0.6 ± 0.2 | 0.6 | 0.8 |

1. Pre-Administration: n=3; Administration: n=3; Recovery-2W: n=1; Recovery-4W: n=1;

2. Compared with the control group: **p*<0.05, ***p*<0.01.

**Table S4.** Coagulation parameters in Beagle dogs from the repeated oral administration of genipin-Males

| **Item** | **Group** | **Pre-Administration** | **Administration** | **Recovery-2W** | **Recovery-4W** |
| --- | --- | --- | --- | --- | --- |
| APTT  (s) | Control | 7.9±0.1 | 8.6 ± 1.2 | 7.3 | 7.4 |
|  | 3 mg/kg | 9.8±0.9 | 11.0 ± 1.3 | 9 | 10.3 |
|  | 9 mg/kg | 9.9±1.8 | 10.3 ± 2.0 | 7.8 | 8.7 |
|  | 27 mg/kg | 9.6±1.8 | 14.8 ± 1.5 ** | 9.6 | 9 |
| PT  (s) | Control | 4.8±0.2 | 5.1 ± 0.4 | 4.7 | 4.7 |
|  | (INR) | 0.43±0.02 | 0.45 ± 0.04 | 0.42 | 0.42 |
|  | 3 mg/kg | 4.7±0.2 | 5.1 ± 0.2 | 5 | 5 |
|  | (INR) | 0.42±0.02 | 0.46 ± 0.01 | 0.45 | 0.45 |
|  | 9 mg/kg | 4.7±0.1 | 5.6 ± 0.4 | 5.1 | 5 |
|  | (INR) | 0.42±0.01 | 0.49 ± 0.03 | 0.46 | 0.45 |
|  | 27 mg/kg | 4.7±0.0 | 5.6 ± 0.2 | 4.9 | 5.1 |
|  | (INR) | 0.42±0.00 | 0.50 ± 0.02 | 0.44 | 0.46 |
| TT  (s) | Control | 13.1±0.7 | 13.4 ± 0.4 | 11.9 | 12.9 |
|  | 3 mg/kg | 13.5±0.6 | 14.2 ± 0.7 | 13.8 | 13.9 |
|  | 9 mg/kg | 13.2±0.5 | 14.5 ± 0.3 | 13.4 | 15.1 |
|  | 27 mg/kg | 13.6±0.8 | 18.2 ± 2.7 ** | 15.1 | 15.2 |
| FIB  (g/L) | Control | 2.114±0.528 | 2.196 ± 0.480 | 1.726 | 1.532 |
|  | 3 mg/kg | 2.183±0.672 | 2.257 ± 0.585 | 1.842 | 2.174 |
|  | 9 mg/kg | 2.021±0.339 | 2.001 ± 0.094 | 2.017 | 2.922 |
|  | 27 mg/kg | 1.734± 0.339 | 2.645 ± 0.436 | 3.221 | 2.805 |

1. Pre-Administration: n=3; Administration: n=3 except for the 27 mg/kg group with n=2 for FIB; Recovery-2W: n=1; Recovery-4W: n=1;

2. Compared with the control group: **p*<0.05, ***p*<0.01.

**Table S5**. Coagulation parameters in Beagle dogs from the repeated oral administration of genipin-Females

| **Item** | **Group** | **Pre-Administration** | | **Administration** | **Recovery-2W** | **Recovery-4W** |
| --- | --- | --- | --- | --- | --- | --- |
| APTT  (s) | Control | | 9.8±0.6 | 9.7 ± 0.4 | 10.9 | 11.1 |
|  | 3 mg/kg | | 10.4±1.2 | 10.1 ± 2.3 | 12.3 | 11.4 |
|  | 9 mg/kg | | 9.1±2.6 | 10.4 ± 3.2 | 8.9 | 8.6 |
|  | 27 mg/kg | | 9.8 ± 1.3 | 14.6 ± 1.1 | 11.3 | 8.1 |
| PT  (s) | Control | | 4.6±0.1 | 5.2 ± 0.2 | 5.1 | 4.9 |
|  | (INR) | | 0.41±0.01 | 0.46 ± 0.01 | 0.46 | 0.44 |
|  | 3 mg/kg | | 4.8±0.1 | 5.2 ± 0.4 | 5.1 | 5 |
|  | (INR) | | 0.43±0.01 | 0.47 ± 0.04 | 0.46 | 0.45 |
|  | 9 mg/kg | | 4.9±0.3 | 5.4 ± 0.6 | 5.4 | 5.4 |
|  | (INR) | | 0.44±0.03 | 0.48 ± 0.05 | 0.48 | 0.48 |
|  | 27 mg/kg | | 4.8±0.2 | 5.2 ± 0.3 | 4.7 | 5.1 |
|  | (INR) | | 0.43 ±0.02 | 0.46 ± 0.03 | 0.42 | 0.46 |
| TT  (s) | Control | | 13.5±0.2 | 12.8 ± 1.3 | 14.0 | 14.8 |
|  | 3 mg/kg | | 13.4±0.6 | 13.2 ± 0.3 | 13.6 | 15.2 |
|  | 9 mg/kg | | 12.7±0.8 | 12.7 ± 1.0 | 11.9 | 13.8 |
|  | 27 mg/kg | | 13.5 ±1.5 | 17.2 ± 1.0 ** | 13.0 | 14.1 |
| FIB  (g/L) | Control | | 1.774±0.561 | 1.328 ± 0.264 | 1.465 | 2.191 |
|  | 3 mg/kg | | 2.130±0.620 | 2.164 ± 0.941 | 2.093 | 2.863 |
|  | 9 mg/kg | | 1.326±0.174 | 1.541 ± 0.320 | 1.624 | 2.572 |
|  | 27 mg/kg | | 1.171 ±0.121 | 1.998 ± 0.208 | 3.048 | 1.783 |

1. Pre-Administration: n=3; Administration: n=3; Recovery-2W: n=1; Recovery-4W: n=1;

2. Compared with the control group: **p*<0.05, ***p*<0.01.

**Table S6.** Clinical chemistry parameters in Beagle dogs from the repeated oral administration of genipin-Males

| **Item** | **Group** | **Pre-Administration** | | **Administration** | **Recovery-2W** | **Recovery-4W** |
| --- | --- | --- | --- | --- | --- | --- |
| ALT  (IU/L) | Control | | 34±7 | 35±9 | 38 | 38 |
|  | 3 mg/kg | | 27±7 | 29±6 | 27 | 23 |
|  | 9 mg/kg | | 34±4 | 61±21 | 32 | 28 |
|  | 27 mg/kg | | 27±8 | 43±14 | 37 | 34 |
| AST  (IU/L) | Control | | 36±3 | 33±4 | 33 | 36 |
|  | 3 mg/kg | | 40±11 | 39±10 | 31 | 26 |
|  | 9 mg/kg | | 37±6 | 43±14 | 27 | 28 |
|  | 27 mg/kg | | 32±7 | 30±7 | 26 | 28 |
| ALP  (IU/L) | Control | | 215±74 | 194±47 | 103 | 94 |
|  | 3 mg/kg | | 206±36 | 208±40 | 170 | 183 |
|  | 9 mg/kg | | 243±30 | 300±39* | 196 | 175 |
|  | 27 mg/kg | | 261±69 | 158±23 | 193 | 180 |
| BUN  (mmol/ L) | Control | | 4.0±0.6 | 3.6±1.0 | 3.7 | 4.0 |
|  | 3 mg/kg | | 4.5±0.8 | 4.8±1.1 | 2.7 | 3.9 |
|  | 9 mg/kg | | 3.8±0.4 | 4.0±0.5 | 3.2 | 3.9 |
|  | 27 mg/kg | | 3.5±0.2 | 4.3±1.0 | 3 | 3.7 |
| CREAT  (µmol/ L) | Control | | 49±2 | 49±3 | 60 | 54 |
|  | 3 mg/kg | | 51±4 | 52±10 | 51 | 51 |
|  | 9 mg/kg | | 49±5 | 40±3 | 48 | 51 |
|  | 27 mg/kg | | 49±4 | 50±14 | 45 | 49 |
| TP  (g/ L) | Control | | 61.9±2.9 | 63.2±4.0 | 64.5 | 60.9 |
|  | 3 mg/kg | | 58.6±1.7 | 60.5±3.0 | 62.6 | 60.0 |
|  | 9 mg/kg | | 60.2±2.7 | 64.0±2.6 | 67.1 | 61.9 |
|  | 27 mg/kg | | 58.7±4.1 | 56.0±1.6* | 60.9 | 59.4 |
| ALB  (g/ L) | Control | | 29.6±0.9 | 29.8±1.1 | 31.5 | 29.5 |
|  | 3 mg/kg | | 28.0±1.5 | 29.2±2.0 | 30.1 | 29.4 |
|  | 9 mg/kg | | 29.4±0.9 | 30.0±0.3 | 30.1 | 28.0 |
|  | 27 mg/kg | | 28.8±1.0 | 25.5±1.1** | 24.6 | 25.1 |
| GLU  (mmol/ L) | Control | | 4.06±0.43 | 3.94±0.50 | 4.53 | 5.44 |
|  | 3 mg/kg | | 4.65±0.64 | 3.99±0.54 | 4.93 | 5.64 |
|  | 9 mg/kg | | 4.42±0.52 | 3.67±0.95 | 4.64 | 5.8 |
|  | 27 mg/kg | | 4.29±0.67 | 3.58±0.50 | 5.08 | 5.68 |
| TB  (μmol/ L) | Control | | 1.44±0.29 | 1.24±0.27 | 1.65 | 1.54 |
|  | 3 mg/kg | | 1.30±0.16 | 0.07±0.06** | 0.86 | 1.66 |
|  | 9 mg/kg | | 1.26±0.30 | 0.00±0.00** | 0.57 | 1.55 |
|  | 27 mg/kg | | 0.99±0.10 | 0.65±0.62 | 1.63 | 0.79 |
| CHOL  (mmol/L) | Control | | 3.85±1.09 | 3.69±0.86 | 3.33 | 3.04 |
|  | 3 mg/kg | | 3.93±1.24 | 3.50±0.78 | 5.79 | 2.87 |
|  | 9 mg/kg | | 4.53±0.42 | 4.51±0.98 | 4.74 | 4.35 |
|  | 27 mg/kg | | 4.22±0.58 | 5.30±0.52 | 4.43 | 4.62 |
| TRIG  (mmol/ L) | Control | | 0.19±0.04 | 0.17±0.03 | 0.26 | 0.38 |
|  | 3 mg/kg | | 0.20±0.01 | 0.19±0.01 | 0.4 | 0.30 |
|  | 9 mg/kg | | 0.20±0.04 | 0.35±0.21 | 0.21 | 0.24 |
|  | 27 mg/kg | | 0.25±0.13 | 0.40±0.12 | 0.42 | 0.46 |
| CK  (IU/L) | Control | | 245±34 | 206±37 | 147 | 200 |
|  | 3 mg/kg | | 250±57 | 252±55 | 195 | 184 |
|  | 9 mg/kg | | 256±11 | 249±70 | 185 | 194 |
|  | 27 mg/kg | | 268±27 | 135±9 | 118 | 143 |
| GGT  (IU/L) | Control | | 3.00±0.00 | 3.00±0.00 | 3.00 | 3.00 |
|  | 3 mg/kg | | 3.67±1.15 | 3.00±1.00 | 2.00 | 5.00 |
|  | 9 mg/kg | | 3.00±0.00 | 2.00±0.00* | 2.00 | 2.00 |
|  | 27 mg/kg | | 2.67±0.58 | 1.67±1.15 | 2.00 | 1.00 |
| HDL-C  (mmol/ L) | Control | | 3.48±0.89 | 3.21±0.71 | 2.98 | 2.81 |
|  | 3 mg/kg | | 3.51±0.88 | 3.24±0.59 | 4.80 | 2.75 |
|  | 9 mg/kg | | 4.01±0.23 | 3.90±0.75 | 4.10 | 3.88 |
|  | 27 mg/kg | | 3.85±0.27 | 4.05±0.23 | 3.66 | 3.99 |
| LDL-C  (mmol/ L) | Control | | 0.27±0.16 | 0.37±0.15 | 0.10 | 0.08 |
|  | 3 mg/kg | | 0.43±0.17 | 0.26±0.10 | 0.66 | 0.03 |
|  | 9 mg/kg | | 0.45±0.26 | 0.53±0.32 | 0.44 | 0.33 |
|  | 27 mg/kg | | 0.35±0.24 | 1.25±0.21** | 0.23 | 0.2 |
| Ca^2+^  (mmol/ L) | Control | | 2.52±0.04 | 2.49±0.02 | 2.55 | 2.46 |
|  | 3 mg/kg | | 2.51±0.32 | 2.50±0.11 | 2.52 | 2.44 |
|  | 9 mg/kg | | 2.55±0.07 | 2.51±0.03 | 2.50 | 2.38 |
|  | 27 mg/kg | | 2.56±0.05 | 2.35±0.06 | 2.41 | 2.38 |
| Na^+^  (mmol/ L) | Control | | 146.5±1.0 | 147.0±0.1 | 134.7 | 135.1 |
|  | 3 mg/kg | | 146.6±0.5 | 147.0±1.6 | 134.2 | 135.5 |
|  | 9 mg/kg | | 146.4±0.4 | 146.9±0.9 | 134.4 | 134.3 |
|  | 27 mg/kg | | 146.4±0.4 | 140.8±6.0 | 134.4 | 134.3 |
| K^+^  (mmol/ L) | Control | | 4.56±0.04 | 4.66±0.15 | 4.05 | 4.32 |
|  | 3 mg/kg | | 4.48±0.38 | 4.81±0.36 | 4.07 | 3.96 |
|  | 9 mg/kg | | 4.70±0.11 | 4.85±0.51 | 4.45 | 4.44 |
|  | 27 mg/kg | | 4.79±0.08 | 4.24±0.68 | 4.26 | 4.38 |
| Cl^-^  (mmol/ L) | Control | | 108.4±1.5 | 111.1±1.5 | 101.6 | 102.9 |
|  | 3 mg/kg | | 108.3±0.2 | 110.1±1.6 | 102.1 | 103.8 |
|  | 9 mg/kg | | 108.3±0.7 | 111.1±1.0 | 102.5 | 102.7 |
|  | 27 mg/kg | | 108.3±0.1 | 104.2±9.5 | 103.6 | 103.1 |

1. Pre-Administration: n=3; Administration: n=3; Recovery-2W: n=1; Recovery-4W: n=1;

2. Compared with the control group: **p*<0.05, ***p*<0.01.

**Table S7.** Clinical chemistry parameters in Beagle dogs from the repeated oral administration of genipin-Females

| **Item** | **Group** | **Pre-Administration** | | **Administration** | **Recovery-2W** | **Recovery-4W** |
| --- | --- | --- | --- | --- | --- | --- |
| ALT  (IU/L) | Control | | 28±6 | 29±5 | 24 | 24 |
|  | 3 mg/kg | | 31±9 | 32±10 | 46 | 36 |
|  | 9 mg/kg | | 30±2 | 29±3 | 31 | 26 |
|  | 27 mg/kg | | 23±4 | 36±21 | 28 | 31 |
| AST  (IU/L) | Control | | 32±1 | 38±10 | 33 | 26 |
|  | 3 mg/kg | | 38±4 | 46±8 | 51 | 30 |
|  | 9 mg/kg | | 35±6 | 32±9 | 28 | 25 |
|  | 27 mg/kg | | 34±3 | 43±5 | 39 | 37 |
| ALP  (IU/L) | Control | | 213±49 | 198±80 | 211 | 148 |
|  | 3 mg/kg | | 251±67 | 262±43 | 212 | 158 |
|  | 9 mg/kg | | 252±26 | 209±48 | 226 | 183 |
|  | 27 mg/kg | | 254±31 | 146±22 | 186 | 197 |
| BUN  (mmol/ L) | Control | | 3.1±0.8 | 3.4±0.7 | 3.8 | 3.8 |
|  | 3 mg/kg | | 3.7±0.7 | 3.9±0.3 | 4.2 | 5.6 |
|  | 9 mg/kg | | 3.9±0.5 | 3.6±0.6 | 3.6 | 4.2 |
|  | 27 mg/kg | | 3. 7±0.5 | 4.1±1.0 | 2.5 | 2.9 |
| CREAT  (µmol/ L) | Control | | 45±3 | 47±6 | 53 | 51 |
|  | 3 mg/kg | | 45±3 | 40±4 | 48 | 46 |
|  | 9 mg/kg | | 46±1 | 40±3 | 57 | 54 |
|  | 27 mg/kg | | 48±3 | 40±2 | 35 | 42 |
| TP  (g/ L) | Control | | 60.5±0.8 | 59.8±2.8 | 62.8 | 58.6 |
|  | 3 mg/kg | | 62.0±0.9 | 65.6±0.7* | 75.7 | 68.1 |
|  | 9 mg/kg | | 58.7±3.1 | 62.0±1.9 | 66.0 | 58.7 |
|  | 27 mg/kg | | 58.8±1.0 | 56.0±6.3 | 60.1 | 62.9 |
| ALB  (g/ L) | Control | | 30.2±0.8 | 30.0±1.3 | 31.3 | 28.1 |
|  | 3 mg/kg | | 30.1±0.4 | 30.6±0.7 | 32.5 | 29.3 |
|  | 9 mg/kg | | 29.8±1.5 | 30.6±1.4 | 30.9 | 27.1 |
|  | 27 mg/kg | | 30.0±0.5 | 25.5±2.9* | 28.0 | 27.4 |
| GLU  (mmol/ L) | Control | | 4.50±0.45 | 4.18±0.73 | 4.48 | 6.22 |
|  | 3 mg/kg | | 4.17±0.37 | 3.79±0.09 | 4.20 | 5.33 |
|  | 9 mg/kg | | 4.36±0.58 | 4.08±0.26 | 5.03 | 5.97 |
|  | 27 mg/kg | | 4.60±0.77 | 3.35±0.75 | 3.68 | 4.54 |
| TB  (μmol/ L) | Control | | 1.46±0.22 | 1.50±0.33 | 1.35 | 1.19 |
|  | 3 mg/kg | | 2.22±0.72 | 1.16±0.29 | 3.27 | 2.29 |
|  | 9 mg/kg | | 1.50±0.35 | 0.00±0.00* | 1.01 | 1.45 |
|  | 27 mg/kg | | 1.43±0.26 | 0.84±1.46 | 2.03 | 0.85 |
| CHOL  (mmol/L) | Control | | 4.79±0.71 | 4.68±0.52 | 3.17 | 4.47 |
|  | 3 mg/kg | | 4.52±0.63 | 4.68±0.70 | 4.64 | 4.08 |
|  | 9 mg/kg | | 4.20±0.54 | 4.56±0.55 | 4.78 | 4.63 |
|  | 27 mg/kg | | 4.05±0.34 | 4.68±0.72 | 3.76 | 4.43 |
| TRIG  (mmol/ L) | Control | | 0.35±0.02 | 0.34±0.12 | 0.23 | 0.60 |
|  | 3 mg/kg | | 0.19±0.09* | 0.23±0.06 | 0.23 | 0.37 |
|  | 9 mg/kg | | 0.36±0.22 | 0.24±0.03 | 0.24 | 0.36 |
|  | 27 mg/kg | | 0.21±0.07* | 0.38±0.09 | 0.28 | 0.35 |
| CK  (IU/L) | Control | | 219±20 | 315±152 | 312 | 175 |
|  | 3 mg/kg | | 295±76 | 416±175 | 238 | 144 |
|  | 9 mg/kg | | 274±90 | 259±86 | 210 | 216 |
|  | 27 mg/kg | | 275±81 | 288±127 | 276 | 183 |
| GGT  (IU/L) | Control | | 3.33±0.58 | 3.00±0.00 | 4.00 | 3.00 |
|  | 3 mg/kg | | 2.67±0.58 | 2.33±0.58 | 4.00 | 3.00 |
|  | 9 mg/kg | | 2.33±0.58 | 1.33±0.58* | 2.00 | 2.00 |
|  | 27 mg/kg | | 2.67±0.58 | 2.33±0.58 | 3.00 | 3.00 |
| HDL-C  (mmol/ L) | Control | | 4.20±0.52 | 4.10±0.35 | 2.85 | 4.91 |
|  | 3 mg/kg | | 4.07±0.45 | 4.16±0.53 | 4.00 | 3.78 |
|  | 9 mg/kg | | 3.84±0.35 | 4.08±0.34 | 4.21 | 4.11 |
|  | 27 mg/kg | | 3.77±0.30 | 3.64±0.50 | 3.31 | 3.99 |
| LDL-C  (mmol/ L) | Control | | 0.55±0.26 | 0.61±0.22 | 0.05 | 0.73 |
|  | 3 mg/kg | | 0.38±0.10 | 0.59±0.23 | 0.23 | 0.19 |
|  | 9 mg/kg | | 0.30±0.23 | 0.46±0.11 | 0.20 | 0.27 |
|  | 27 mg/kg | | 0.34±0.17 | 1.05±0.43 | 0.11 | 0.25 |
| Ca^2+^  (mmol/ L) | Control | | 2.56±0.01 | 2.47±0.02 | 2.47 | 2.41 |
|  | 3 mg/kg | | 2.50±0.03 | 2.44±0.07 | 2.62 | 2.43 |
|  | 9 mg/kg | | 2.56±0.07 | 2.50±0.08 | 2.56 | 2.42 |
|  | 27 mg/kg | | 2.48±0.05 | 2.22±0.06** | 2.37 | 2.38 |
| Na^+^  (mmol/ L) | Control | | 146.6±0.6 | 146.7±0.6 | 135.0 | 135.3 |
|  | 3 mg/kg | | 146.5±2.1 | 146.0±2.0 | 134.8 | 135.1 |
|  | 9 mg/kg | | 146.3±0.6 | 146.6±1.7 | 135.1 | 135.4 |
|  | 27 mg/kg | | 146.5±1.0 | 142.9±6.1 | 135.8 | 134.5 |
| K^+^  (mmol/ L) | Control | | 4.60±0.13 | 4.47±0.16 | 3.95 | 4.28 |
|  | 3 mg/kg | | 4.62±0.21 | 4.50±0.20 | 3.61 | 3.99 |
|  | 9 mg/kg | | 4.52±0.37 | 4.57±0.12 | 3.67 | 4.17 |
|  | 27 mg/kg | | 4.66±0.07 | 4.66±0.68 | 4.35 | 4.35 |
| Cl^-^  (mmol/ L) | Control | | 107.8±1.2 | 109.8±0.7 | 104.5 | 104.1 |
|  | 3 mg/kg | | 108.6±1.5 | 109.3±1.2 | 104.7 | 105.2 |
|  | 9 mg/kg | | 107.7±1.4 | 109.1±1.1 | 104.0 | 105.4 |
|  | 27 mg/kg | | 109.6±1.0 | 108.2±9.9 | 107.0 | 104.1 |

1. Pre-Administration: n=3; Administration: n=3; Recovery-2W: n=1; Recovery-4W: n=1;

2. Compared with the control group: **p*<0.05, ***p*<0.01.

**Table S8.** ECGs in Beagle dogs from the repeated oral administration of genipin-Males

| **Item** | | **Group** | **Pre-Administration** | | | | **Administration** | | **Recovery-2W** | | **Recovery-4W** |
| --- | --- | --- | --- | --- | --- | --- | --- | --- | --- | --- | --- |
| P  （1/100mv） | | Control | | | 18.3±4.9 | | 28.3±5.9 | | 16 | | -1 |
|  |  | 3 mg/kg | | | 9.7±13.0 | | 17.0±8.7 | | 31 | | 35 |
|  |  | 9 mg/kg | | | 20.7±0.6 | | 29.7±12.5 | | 26 | | 40 |
|  |  | 27 mg/kg | | | 17.3±9.6 | | 29.0±19.1 | | 17 | | 30 |
| PR  （ms） | | Control | | | 106.3±12.7 | | 103.0±6.2 | | 116 | | 114 |
|  |  | 3 mg/kg | | | 106.3±19.7 | | 94.3±3.2 | | 96 | | 100 |
|  |  | 9 mg/kg | | | 96.7±6.0 | | 109.0±4.6△ | | 90 | | 94 |
|  |  | 27 mg/kg | | | 105.3±1.5 | | 95.3±4.7△△ | | 98 | | 120 |
| QRS  （ms） | | Control | | | 51.0±7.6 | | 52.0±9.8 | | 52 | | 61 |
|  |  | 3 mg/kg | | | 59.7±10.1 | | 49.3±10.1 | | 58 | | 63 |
|  |  | 9 mg/kg | | | 43.3±4.5 | | 44.7±3.8 | | 48 | | 56 |
|  |  | 27 mg/kg | | | 48.3±8.4 | | 50.3±10.2 | | 45 | | 45 |
| QT  （ms） | | Control | | | 238.7±53.7 | | 219.3±22.4 | | 250 | | 262 |
|  |  | 3 mg/kg | | | 266.3±49.5 | | 214.3±10.4 | | 223 | | 208 |
|  |  | 9 mg/kg | | | 230.0±30.3 | | 230.0±23.9 | | 199 | | 202 |
|  |  | 27 mg/kg | | | 206.7±23.9 | | 239.7±23.2 | | 219 | | 186 |
| T  （1/100mv） | | Control | | | 8.3±15.4 | | 8.0±15.9 | | 19 | | 5 |
|  |  | 3 mg/kg | | | -12.3±85.4 | | -12.0±32.2 | | -54 | | -45 |
|  |  | 9 mg/kg | | | 20.0±7.6 | | 36.7±30.7 | | 44 | | -29 |
|  |  | 27 mg/kg | | | 21.7±32.3 | | 29.3±55.1 | | 29 | | 97 |
| ST  （1/100mv） | | Control | | | 0.7±3.2 | | 1.3±3.5 | | 5 | | 22 |
|  |  | 3 mg/kg | | | -2.0±13.9 | | -5.7±2.1 | | -8 | | 2 |
|  |  | 9 mg/kg | | | -3.3±3.2 | | -3.3±5.5 | | -6 | | -13 |
|  |  | 27 mg/kg | | | -5.0±2.6 | | -11.7±6.1** | | -7 | | -14 |
| HR  （bpm） | Control | | | 104.0±28.4 | | 88.3±25.1 | | 82 | | 100 | |
|  | 3 mg/kg | | | 103.0±43.7 | | 93.3±32.5 | | 133 | | 129 | |
|  | 9 mg/kg | | | 110.0±13.9 | | 91.3±1.5△ | | 111 | | 80 | |
|  | 27 mg/kg | | | 121.3±28.6 | | 114.3±22.7 | | 151 | | 121 | |

1. Pre-Administration: n=3; Administration: n=3; Recovery-2W: n=1; Recovery-4W: n=1;

2. Compared with the control group: **p*<0.05, ***p*<0.01;

3. Compared with the pre-administration baselines: △△*p*<0.01, △*p*<0.05.

**Table S9.** ECGs in Beagle dogs from the repeated oral administration of genipin-Females

| **Item** | | **Group** | **Pre-Administration** | | | | **Administration** | | **Recovery-2W** | | **Recovery-4W** |
| --- | --- | --- | --- | --- | --- | --- | --- | --- | --- | --- | --- |
| P  （1/100mv） | | Control | | | 17.0±14.2 | | 45.7±25.0 | | 30 | | 5 |
|  |  | 3 mg/kg | | | 19.0±5.3 | | 34.3±11.6 | | 23 | | 43 |
|  |  | 9 mg/kg | | | 19.3±8.6 | | 29.3±23.6 | | 30 | | 3 |
|  |  | 27 mg/kg | | | 14.7±22.5 | | 24.0±8.2 | | 15 | | 17 |
| PR  （ms） | | Control | | | 123.0±8.0 | | 101.7±12.7 | | 106 | | 127 |
|  |  | 3 mg/kg | | | 106.0±13.9 | | 116.0±18.2 | | 96 | | 95 |
|  |  | 9 mg/kg | | | 111.7±9.0 | | 101.0±7.0 | | 108 | | 120 |
|  |  | 27 mg/kg | | | 122.7±17.6 | | 108.7±13.2 | | 87 | | 100 |
| QRS  （ms） | | Control | | | 57.3±8.1 | | 50.0±5.0 | | 49 | | 58 |
|  |  | 3 mg/kg | | | 60.7±2.5 | | 53.3±9.0 | | 58 | | 61 |
|  |  | 9 mg/kg | | | 56.3±3.8 | | 51.0±7.6 | | 53 | | 61 |
|  |  | 27 mg/kg | | | 52.7±2.5 | | 57.0±3.6 | | 42 | | 49 |
| QT  （ms） | | Control | | | 239.3±17.7 | | 208.7±3.8 | | 207 | | 217 |
|  |  | 3 mg/kg | | | 285.0±45.6 | | 203.7±24.8△ | | 196 | | 208 |
|  |  | 9 mg/kg | | | 242.0±24.3 | | 243.0±54.7 | | 229 | | 234 |
|  |  | 27 mg/kg | | | 221.7±12.7 | | 270.7±93.9 | | 214 | | 192 |
| T  （1/100mv） | | Control | | | 42.0±48.5 | | 35.7±54.3 | | 37 | | 82 |
|  |  | 3 mg/kg | | | 18.3±4.0 | | 35.0±33.8 | | 14 | | 47 |
|  |  | 9 mg/kg | | | 24.3±26.5 | | 11.3±26.5 | | 20 | | 112 |
|  |  | 27 mg/kg | | | 29.7±20.3 | | 30.3±51.1 | | 12 | | 72 |
| ST  （1/100mv） | | Control | | | 0.3±10.2 | | 0.3±9.1 | | 8 | | 29 |
|  |  | 3 mg/kg | | | 2.3±3.2 | | -0 .3±7.2 | | 0 | | 8 |
|  |  | 9 mg/kg | | | -1.7±9.0 | | 0.7±13.0 | | 2 | | 22 |
|  |  | 27 mg/kg | | | -1.7±5.9 | | -12.3±10.4 | | -2 | | -1 |
| HR  （bpm） | Control | | | 93.0±32.0 | | 95.0±14.8 | | 102 | | 90 | |
|  | 3 mg/kg | | | 116.0±36.0 | | 128.7±37.8 | | 140 | | 152 | |
|  | 9 mg/kg | | | 113.7±28.5 | | 95.7±5.1 | | 116 | | 103 | |
|  | 27 mg/kg | | | 106.0±13.0 | | 105.0±25.0 | | 117 | | 120 | |

1. Pre-Administration: n=3; Administration: n=3; Recovery-2W: n=1; Recovery-4W: n=1;

2. Compared with the control group: **p*<0.05, ***p*<0.01;

3. Compared with the pre-administration baselines: △△*p*<0.01, △*p*<0.05.

**Table S10.** Pathological alterations in Beagle dogs from the repeated oral administration of genipin-Males

| **Animal no.** | **Dose (****mg/kg)** | **Administration period** | | **Recovery period** | |
| --- | --- | --- | --- | --- | --- |
|  |  | **Gross pathology observations** | **Histopathological examination** | **Gross pathology observations** | **Histopathological examination** |
| 1 | 0 | - | - | - | - |
| 2 |  | - | Pituitary Rathke's cleft cyst (+) Thymic atrophy (±) | - | - |
| 3 |  | - | - | - | Hepatocellular glycogen accumulation: (±) Hepatic focal necrosis or microgranulomas: (±) |
| 7 | 3 | Bilateral testes and prostate: reduced size | Testicular atrophy with decreased spermatogenic cells (±) Epididymal atrophy (+) with absent intraductal sperm Prostatic atrophy (++) with interstitial fibrosis (+) | - | - |
| 8 |  | - | Focal dilation of individual glandular ducts in the duodenal mucosa (±) | - | - |
| 9 |  | - | - | - | - |
| 13 | 9 | - | Focal granulomatous inflammatory lesions in the lung (±) Pituitary Rathke's cleft cyst (+) | - | - |
| 14 |  | Prostate: reduced size | Prostatic atrophy (++) and interstitial fibrosis (+) | - | - |
| 15 |  | - | - | Duodenal mucosa: pinhead-sized white defects | Pituitary Rathke's cleft cyst: (+) |
| 19 | 27 | Liver, bilateral kidneys, salivary glands, and mesenteric lymph nodes: blue discoloration Bilateral testes, epididymides, and prostate: reduced size Gallbladder mucosa: erythema Gastric pyloric mucosa: pallor | Periportal hepatocellular swelling and vacuolar degeneration(++) Gastric mucosal regenerative changes (±) and focal erosion (±) Gallbladder mucosal epithelial hyperplasia (+) and lamina propria inflammatory cell infiltration (+) Focal glomerular and interstitial hypoplasia in the left kidney (±) Testicular atrophy with decreased spermatogenic cells (+) epididymal atrophy (+) with absent intraductal sperm; prostatic atrophy (++) with interstitial fibrosis (+) Thymic atrophy (++) Thoracic bone marrow: decreased hematopoietic cells with increased adipose tissue | - | - |
| 20 |  | Liver, bilateral kidneys, salivary glands, and mesenteric lymph nodes: blue discoloration Bilateral testes, epididymides, and prostate: reduced size Duodenal mucosa: several pinhead-sized white defects | Periportal hepatocellular swelling (+) Portal tract vascular endothelial hyperplasia (±) Gastric mucosal regenerative changes (+) and focal erosion (±) Focal autolysis of gallbladder mucosal epithelium (±) Testicular atrophy with decreased spermatogenic cells (+) Epididymal atrophy (+) with absent intraductal sperm Prostatic atrophy (++) with interstitial fibrosis (+) Thymic atrophy (++) Thoracic bone marrow: decreased hematopoietic cells with increased adipose tissue (++) | - | - |
| 21 |  | Liver, bilateral kidneys, salivary glands, and mesenteric lymph nodes: blue discoloration | - | Liver, bilateral kidneys, and salivary glands: slightly blue discoloration Thymus: reduced size Bilateral testes and epididymides: reduced size | Pulmonary hemorrhage: (++) Hepatocellular glycogen accumulation: (++) Hepatic focal necrosis or microgranulomas: (+) Proliferation of vascular endothelial cells and fibroblasts in hepatic portal area: (±) Renal tubular epithelial degeneration in deep cortex of left kidney: (±) Focal testicular atrophy: (+) Epididymal atrophy: (+); no sperm detected in the ducts Prostatic atrophy: (±) Thymic atrophy: (+) |

**Table S11.** Pathological Alterations in Beagle dogs from the repeated oral administration of genipin-Females

| **Animal no.** | **Dose (mg/kg)** | **Administration period** | | **Recovery period** | |
| --- | --- | --- | --- | --- | --- |
|  |  | **Gross pathology observations** | **Histopathological examination** | **Gross pathology observations** | **Histopathological examination** |
| 4 | 0 | - | Thymic atrophy (±) | - | - |
| 5 |  | - | Hepatic microgranuloma (±) | - | - |
| 6 |  | - | - | Bilateral lungs: scattered white macules Uterus: enlargement with firm texture Cervical canal: dilation, with a 1×1 cm2 firm white mass | Hepatocellular glycogen accumulation: (±) Hepatic focal necrosis or microgranulomas: (±) Mass in cervical canal: identified as cervical polyp |
| 10 | 3 | Duodenal mucosa: erythema | - | - | - |
| 11 |  | - | Hepatic microgranuloma (±) Focal autolysis of gastric mucosa (±) Endometrial glandular hyperplasia (+) Myometrial hypertrophy (+) | - | - |
| 12 |  | - | - | Bilateral ovaries: reduced size | Hepatocellular glycogen accumulation: (±) Hepatic focal necrosis or microgranulomas: (±) Inflammatory cell infiltration in salivary gland interstitium: (±) |
| 16 | 9 | - | Pituitary Rathke's cleft cyst (+) | - | - |
| 17 |  | Liver and bilateral kidneys: blue discoloration Bilateral lung margins: visible white plaques | Focal dilation of individual glandular ducts in the duodenal mucosa (±) | - | - |
| 18 |  | - | - | - | Focal dilation of individual glandular ducts in duodenal mucosa: (±) Focal interstitial fibrosis and renal tubular dilation in left kidney: (±) |
| 22 | 27 | Liver, bilateral kidneys, salivary glands, and mesenteric lymph nodes: blue discoloration Uterus: reduced size Thymus: reduced size Splenic surface: visible white plaques | Periportal hepatocellular swelling (+) Portal tract vascular endothelial hyperplasia (±) Focal hyperplasia of gallbladder mucosal epithelium (±) Focal autolysis of gallbladder mucosal epithelium (±) Renal tubular epithelial swelling and vacuolar degeneration (+) Uterine atrophy (+) Follicular differentiation disorder (++) Pituitary Rathke's cleft cyst (+) Thymic atrophy (++) Thoracic bone marrow: decreased hematopoietic cells with increased adipose tissue (++) Focal capsular degeneration and thickening of the spleen (+) | - | - |
| 23 |  | Liver, bilateral kidneys, salivary glands, and mesenteric lymph nodes: blue discoloration Uterus: reduced size Duodenal mucosa: several pinhead-sized white defects | Periportal hepatocellular swelling (+) Portal tract vascular endothelial hyperplasia (±) Focal hyperplasia of superficial gastric gland ducts (±) Focal hyperplasia of gallbladder mucosal epithelium (±) Uterine atrophy (+) Pituitary Rathke's cleft cyst (+) Thymic atrophy (++) Thoracic bone marrow: decreased hematopoietic cells with increased adipose tissue (++) | - | - |
| 24 |  | Liver, bilateral kidneys, salivary glands, and mesenteric lymph nodes: blue discoloration | - | Liver, bilateral kidneys, and salivary glands: slightly blue discoloration Thymus: reduced size Uterus and bilateral ovaries: reduced size Duodenal mucosa: one pinhead-sized white defect | Pacinian corpuscles in sciatic nerve: (±) Hepatocellular glycogen accumulation: (++) Focal necrosis or microgranulomas in liver: (+) Proliferation of vascular endothelial cells and fibroblasts in hepatic portal area: (±) Uterine atrophy: (+) Follicular differentiation disorder: (++) Pituitary Rathke's cleft cyst: (+) |

**Table S12.** Histopathological profile in Beagle dogs from the repeated oral administration of genipin

| **Organ/Tissue** | **Histological changes** | **Administration period** | | | | | | | | **Recovery period** | | | | | | | |
| --- | --- | --- | --- | --- | --- | --- | --- | --- | --- | --- | --- | --- | --- | --- | --- | --- | --- |
|  |  | **0** | | **3 mg/kg** | | **9 mg/kg** | | **27 mg/kg** | | **0** | | **3 mg/kg** | | **9 mg/kg** | | **27 mg/kg** | |
|  |  | ♂ | ♀ | ♂ | ♀ | ♂ | ♀ | ♂ | ♀ | ♂ | ♀ | ♂ | ♀ | ♂ | ♀ | ♂ | ♀ |
| Pituitary | Rathke's cleft cyst | (1/2)  [1] | (0/2)  [0] | (0) | (0) | (1/2)  [1] | (1/2)  [1] | (0) | (2/2)  [2] | (0) | (0) | (0) | (0) | (1/1)  [2] | (0) | 0  [0] | (1/1)  [2] |
| Thymus | Thymic atrophy | (1/2)  [0.5] | (1/2)  [0.5] | (0) | (0) | (0) | (0) | (2/2)  [3] | (2/2)  [3] | (0) | (0) | (0) | (0) | (0) | (0) | (1/1)  [2] | (0) |
| Thoracic bone marrow | Decreased hematopoietic cells with increased adipose tissue | (0) | (0) | (0) | (0) | (0) | (0) | (2/2)  [3] | (2/2)  [3] | (0) | (0) | (0) | (0) | (0) | (0) | (0) | (0) |
| Liver | Hepatic focal necrosis or microgranulomas | (0) | (1/2)  [0.5] | (0) | (1/2)  [0.5] | (0) | (0) | (0) | (0) | (1/1)  [1.5] | (1/1)  [1.5] | (0) | (1/1)  [1.5] | (0) | (0) | (1/1)  [1.5] | (1/1)  [1.5] |
|  | Periportal hepatocellular swelling and vacuolar degeneration | (0) | (0) | (0) | (0) | (0) | (0) | (2/2)  [2.5] | (2/2)  [2] | (0) | (0) | (0) | (0) | (0) | (0) | (0) | (0) |
|  | Proliferation of vascular endothelial cells and fibroblasts in hepatic portal area | (0) | (0) | (0) | (0) | (0) | (0) | (1/2)  [0.5] | (2/2)  [1] | (0) | (0) | (0) | (0) | (0) | (0) | (1/1)  [2] | (1/1)  [1] |
|  | Hepatocellular glycogen accumulation | (0) | (0) | (0) | (0) | (0) | (0) | (0) | (0) | (1/1)  [2] | (1/1)  [2] | (0) | (1/1)  [2] | (0) | (0) | (1/1)  [1] | (1/1)  [2] |
| Testis | Testicular atrophy with decreased spermatogenic cells | (1/2)  [1] | (0) | (1/2)  [1] | (0) | (0) | (0) | (2/2)  [2] | (0) | (0) | (0) | (0) | (0) | (0) | (0) | (1/1)  [2] | (0) |
| Epididymis | Epididymal atrophy | (1/2)  [0.5] | (0) | (1/2)  [1] | (0) | (0) | (0) | (2/2)  [2] | (0) | (0) | (0) | (0) | (0) | (0) | (0) | (1/1)  [2] | (0) |
|  | Absent intraductal sperm | (0) | (0) | (1/2) | (0) | (0) | (0) | (2/2) | (0) | (0) | (0) | (0) | (0) | (0) | (0) | (1/1) | (0) |
| Prostate | Prostatic atrophy | (0) | (0) | (1/2)  [1.5] | (0) | (1/2)  [1.5] | (0) | (2/2)  [3] | (0) | (0) | (0) | (0) | (0) | (0) | (0) | (1/1)  [1] | (0) |
|  | Interstitial fibrosis | (0) | (0) | (1/2)  [1] | (0) | (1/2)  [1] | (0) | (2/2)  [2] | (0) | (0) | (0) | (0) | (0) | (0) | (0) | (0) | (0) |
| Duodenum | Focal dilation of individual glandular ducts in the duodenal mucosa | (0) | (0) | (1/2)  [0.5] | (0) | (0) | (1/2)  [0.5] | (0) | (0) | (0) | (0) | (0) | (0) | (0) | (1/1)  [1] | (0) | (0) |
| Stomach | Gastric mucosal regenerative changes | (0) | (0) | (0) | (0) | (0) | (0) | (2/2)  [1.5] | (0) | (0) | (0) | (0) | (0) | (0) | (0) | (0) | (0) |
|  | Focal erosion | (0) | (0) | (0) | (0) | (0) | (0) | (2/2)  [1] | (0) | (0) | (0) | (0) | (0) | (0) | (0) | (0) | (0) |
|  | Focal hyperplasia of superficial gastric gland ducts | (0) | (0) | (0) | (0) | (0) | (0) | (2/2)  [0.5] | (0) | (0) | (0) | (0) | (0) | (0) | (0) | (0) | (0) |
|  | Focal autolysis of gastric mucosa | (0) | (0) | (1/2)  [0.5] | (0) | (0) | (0) | (0) | (0) | (0) | (0) | (0) | (0) | (0) | (0) | (0) | (0) |
| Uterus | Uterine atrophy | (0) | (0) | (0) | (0) | (0) | (0) | (0) | (2/2)  [2] | (0) | (0) | (0) | (0) | (0) | (0) | (0) | (1/1)  [2] |
|  | Endometrial glandular hyperplasia | (0) | (0) | (0) | (1/2)  [1] | (0) | (0) | (0) | (0) | (0) | (0) | (0) | (0) | (0) | (0) | (0) | (0) |
|  | Myometrial hypertrophy | (0) | (0) | (0) | (1/2)  [1] | (0) | (0) | (0) | (0) | (0) | (0) | (0) | (0) | (0) | (0) | (0) | (0) |
|  | Cervical polyp | (0) | (0) | (0) | (0) | (0) | (0) | (0) | (0) | (0) | (1/1) | (0) | (0) | (0) | (0) | (0) | (0) |
| Follicle | Follicular differentiation disorder | (0) | (0) | (0) | (0) | (0) | (0) | (0) | (1/2)  [1.5] | (0) | (0) | (0) | (0) | (0) | (0) | (0) | (1/1)  [3] |
| Lung | Pulmonary hemorrhage | (0) | (0) | (0) | (0) | (0) | (0) | (0) | (0) | (0) | (0) | (0) | (0) | (0) | (0) | (1/1)  [3] | (0) |
|  | Focal granulomatous inflammatory lesions | (0) | (0) | (0) | (0) | (0) | (0) | (0) | (1/2)  [0.5] | (0) | (0) | (0) | (0) | (0) | (0) | (0) | (0) |
| Gallbladder | Gallbladder mucosal epithelial hyperplasia | (0) | (0) | (0) | (0) | (0) | (0) | (1/2)  [1] | (2/2)  [1] | (0) | (0) | (0) | (0) | (0) | (0) | (0) | (0) |
|  | Lamina propria inflammatory cell infiltration | (0) | (0) | (0) | (0) | (0) | (0) | (1/2)  [1] | (0) | (0) | (0) | (0) | (0) | (0) | (0) | (0) | (0) |
|  | Focal autolysis of gallbladder mucosal epithelium | (0) | (0) | (0) | (0) | (0) | (0) | (1/2)  [0.5] | (1/2)  [0.5] | (0) | (0) | (0) | (0) | (0) | (0) | (0) | (0) |
| Kidney | Focal glomerular and interstitial hypoplasia in the left kidney | (0) | (0) | (0) | (0) | (0) | (0) | (1/2)  [0.5] | (0) | (0) | (0) | (0) | (0) | (0) | (0) | (0) | (0) |
|  | Renal tubular epithelial degeneration in deep cortex of left kidney | (0) | (0) | (0) | (0) | (0) | (0) | (0) | (0) | (0) | (0) | (0) | (0) | (0) | (0) | (1/1)  [1] | (0) |
|  | Renal tubular epithelial swelling and vacuolar degeneration | (0) | (0) | (0) | (0) | (0) | (0) | (0) | (1/2)  [1] | (0) | (0) | (0) | (0) | (0) | (0) | (0) | (0) |
|  | Focal interstitial fibrosis | (0) | (0) | (0) | (0) | (0) | (0) | (0) | (0) | (0) | (0) | (0) | (0) | (0) | (1/1)  [1] | (0) | (0) |
|  | Renal tubular dilation | (0) | (0) | (0) | (0) | (0) | (0) | (0) | (0) | (0) | (0) | (0) | (0) | (0) | (1/1)  [1] | (0) | (0) |
| Spleen | Focal capsular degeneration and thickening of the spleen | (0) | (0) | (0) | (0) | (0) | (0) | (0) | (1/2)  [1] | (0) | (0) | (0) | (0) | (0) | (0) | (0) | (0) |
| Salivary gland | Inflammatory cell infiltration in salivary gland interstitium | (0) | (0) | (0) | (0) | (0) | (0) | (0) | (0) | (0) | (0) | (0) | (1/1)  [1] | (0) | (0) | (0) | (0) |
| Sciatic nerve | Pacinian corpuscles in sciatic nerve | (0) | (0) | (0) | (0) | (0) | (0) | (0) | (0) | (0) | (0) | (0) | (0) | (0) | (0) | (0) | (1/1)  [1] |

Numbers in round parentheses ( ): incidences of lesions in each organ/tissue per group;

Numbers in square brackets [ ]: group‑average severity scores; Higher scores indicate greater severity.

**Table S13.** Organ indices of Beagle dogs at the termination of repeated genipin oral administration-Males

| **Organ Index** | **Control** | **3 mg/kg** | **9 mg/kg** | **27 mg/kg** |
| --- | --- | --- | --- | --- |
| hepatic index | 0.768±0.117 | 0.864±0.083 | 0.757±0.070 | 0.685±0.102 |
| hepatic index | 2.669±0.112 | 2.651±0.214 | 3.033±0.276 | 3.768±0.469 |
| splenic index | 0.298±0.052 | 0.281±0.054 | 0.318±0.030 | 0.322±0.029 |
| pulmonary index | 0.816±0.010 | 0.820±0.040 | 0.834±0.008 | 0.984±0.021 |
| renal index | 0.452±0.020 | 0.479±0.060 | 0.499±0.031 | 0.589±0.033 |
| adrenal index | 0.013±0.000 | 0.012±0.001 | 0.017±0.003 | 0.024±0.008 |
| thymic index | 0.072±0.003 | 0.115±0.035 | 0.087±0.013 | 0.082±0.047 |
| cerebral index | 0.855±0.016 | 0.870±0.042 | 0.823±0.088 | 1.085±0.002 |
| testicular index | 0.171±0.015 | 0.123±0.077 | 0.146±0.005 | 0.060±0.015 |
| epididymal index | 0.033±0.006 | 0.030±0.009 | 0.026±0.003 | 0.018±0.000 |
| pituitary index | 0.001±0.000 | 0.001±0.000 | 0.001±0.000 | 0.001±0.000 |

n=2

**Table S14.** Organ indices of Beagle Dogs at the termination of the recovery period following repeated oral genipin administration-Males (g/100g)

| **Organ Index** | **Control** | **3 mg/kg** | **9 mg/kg** | **27 mg/kg** |
| --- | --- | --- | --- | --- |
| hepatic index | 0.733 | 0.869 | 0.748 | 0.824 |
| hepatic index | 2.617 | 2.789 | 2.991 | 3.169 |
| splenic index | 0.267 | 0.308 | 0.28 | 0.302 |
| pulmonary index | 0.861 | 0.915 | 0.788 | 1.096 |
| renal index | 0.441 | 0.458 | 0.511 | 0.537 |
| adrenal index | 0.009 | 0.014 | 0.01 | 0.013 |
| thymic index | 0.174 | 0.137 | 0.091 | 0.098 |
| cerebral index | 0.729 | 0.758 | 0.761 | 0.935 |
| testicular index | 0.103 | 0.183 | 0.158 | 0.097 |
| epididymal index | 0.024 | 0.031 | 0.03 | 0.025 |
| pituitary index | 0.001 | 0.001 | 0.001 | 0.001 |

n=1

**Table S15.** Organ indices of Beagle dogs at the termination of repeated oral genipin administration-Females

| **Organ Index** | **Control** | **3 mg/kg** | **9 mg/kg** | **27 mg/kg** |
| --- | --- | --- | --- | --- |
| hepatic index | 0.788±0.001 | 0.783±0.147 | 0.804±0.051 | 0.817±0.045 |
| hepatic index | 2.580±0.116 | 2.934±0.000 | 2.602±0.088 | 3.279±0.018 |
| splenic index | 0.277±0.063 | 0.364±0.087 | 0.325±0.075 | 0.395±0.035 |
| pulmonary index | 0.932±0.091 | 0.930±0.122 | 0.805±0.004 | 0.993±0.070 |
| renal index | 0.502±0.019 | 0.489±0.021 | 0.442±0.030 | 0.512±0.018 |
| adrenal index | 0.014±0.003 | 0.017±0.001 | 0.018±0.004 | 0.026±0.003 |
| thymic index | 0.066±0.003 | 0.072±0.032 | 0.115±0.004 | 0.041±0.012 |
| cerebral index | 0.983±0.129 | 0.967±0.112 | 0.936±0.105 | 1.341±0.012 |
| uterine index | 0.044±0.025 | 0.103±0.106 | 0.037±0.009 | 0.017±0.004 |
| ovarian index | 0.010±0.004 | 0.015±0.008 | 0.015±0.008 | 0.015±0.002 |
| pituitary index | 0.001±0.000 | 0.001±0.000 | 0.001±0.000 | 0.001±0.000 |

n=2

**Table S16.** Organ indices of Beagle Dogs at the termination of the recovery period following repeated oral genipin administration-Females (g/100g)

| **Organ Index** | **Control** | **3 mg/kg** | **9 mg/kg** | **27 mg/kg** |
| --- | --- | --- | --- | --- |
| hepatic index | 0.781 | 0.683 | 0.833 | 0.92 |
| hepatic index | 3.007 | 2.658 | 3.056 | 3.31 |
| splenic index | 0.268 | 0.377 | 0.307 | 0.387 |
| pulmonary index | 0.774 | 0.934 | 0.86 | 0.813 |
| renal index | 0.422 | 0.403 | 0.476 | 0.519 |
| adrenal index | 0.013 | 0.013 | 0.013 | 0.015 |
| thymic index | 0.132 | 0.132 | 0.154 | 0.076 |
| cerebral index | 0.739 | 0.821 | 0.757 | 0.957 |
| uterine index | 0.218 | 0.043 | 0.167 | 0.044 |
| ovarian index | 0.013 | 0.011 | 0.015 | 0.009 |
| pituitary index | 0.001 | 0.001 | 0.001 | 0.001 |

n=1
